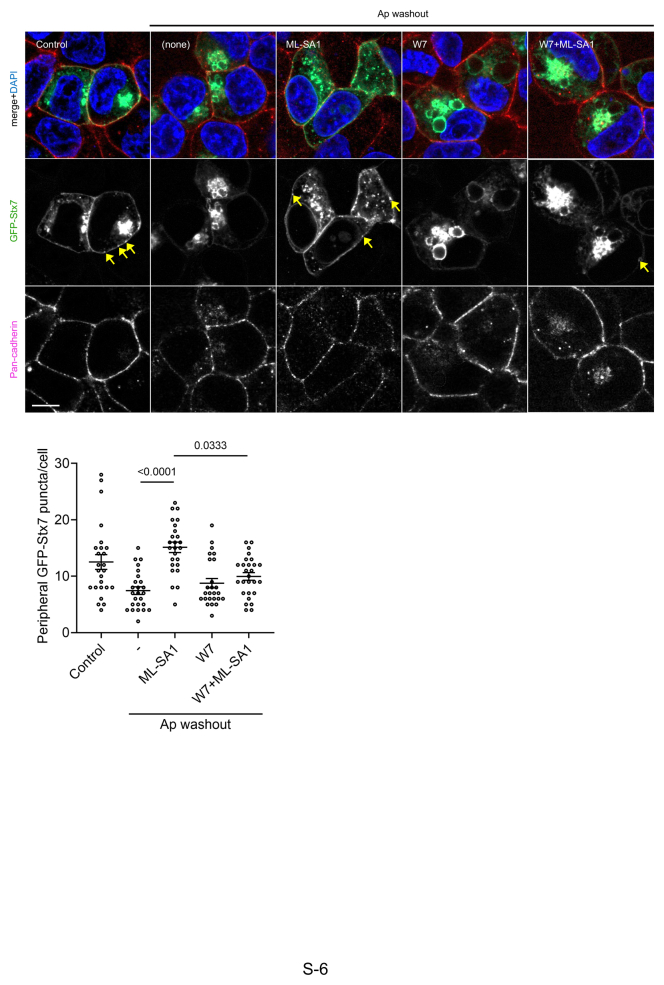# Lysosomal activity depends on TRPML1-mediated Ca^2+^ release coupled to incoming vesicle fusions

**DOI:** 10.1016/j.jbc.2024.107911

**Published:** 2024-10-19

**Authors:** Arindam Bhattacharjee, Hussein Abuammar, Gábor Juhász

**Affiliations:** 1 Institute of Genetics, MTA Lendület Lysosomal Degradation Research Group, HUN-REN BRC Szeged, Szeged, Hungary; 2 Biology Doctoral School, University of Szeged, Szeged, Hungary; 3 Department of Anatomy, Cell and Developmental Biology, ELTE, Budapest, Hungary

**Keywords:** lysosomal acidification, autophagy, ion channel, SNARE proteins, membrane fusion

## Abstract

The lysosomal cation channel TRPML1/MCOLN1 facilitates autophagic degradation during amino acid starvation based on studies involving long-term TRMPL1 modulation. Here we show that lysosomal activation (more acidic pH and higher hydrolase activity) depends on incoming vesicle fusions. We identify an immediate, calcium-dependent role of TRPML1 in lysosomal activation through promoting autophagosome-lysosome fusions and lysosome acidification within 10 to 20 min of its pharmacological activation. Lysosomes also become more fusion competent upon TRPML1 activation *via* increased transport of lysosomal SNARE proteins syntaxin 7 and VAMP7 by SNARE carrier vesicles. We find that incoming vesicle fusion is a prerequisite for lysosomal Ca^2+^ efflux that leads to acidification and hydrolytic enzyme activation. Physiologically, the first vesicle fusions likely trigger generation of the phospholipid PI(3,5)P_2_ that activates TRPML1, and allosteric TRPML1 activation in the absence of PI(3,5)P_2_ restores autophagosome-lysosome fusion and rescues abnormal SNARE sequestration within lysosomes. We thus identify a prompt role of TRPML1-mediated calcium signaling in lysosomal fusions, activation, and SNARE trafficking.

Transient receptor potential cation channels, mucolipin subfamily (TRPMLs) are a group of nonselective cation channels present in late endosomal and lysosomal membranes. Their roles have been implicated in lysosome biogenesis, endosome-lysosome fusion, exocytosis, and lysosomal membrane transport, respectively (1, 2, 3). The lipid storage disorders mucolipidosis type IV and Niemann-Pick type C are characterized by loss of TRPML1 function (4). On the contrary, TRPML1 overexpression drives cell proliferation in HRAS-mutant tumors (5). Recently, growing evidence points to autophagy-related functions of this protein (6). First, TRPML1 current is increased during amino acid starvation (7). Next, *Trpml* mutant *Drosophila* cells show large-scale accumulation of autolysosomes that manage to fuse with autophagosomes, but contain undegraded cargo (8). At a cellular level, dynein-directed lysosome motility, a prerequisite for autophagosome-lysosome fusion, is also regulated by TRPML1-released Ca^2+^ that activates the dynein- and TRPML1 interacting protein ALG-2. TRPML1 inhibition can thus also interfere with proper lysosome positioning and autophagic fusions during starvation (9). A role of TRPML1-released Ca^2+^ in autophagosome biogenesis has also been proposed: acute stimulation of TRPML1 activated the upstream autophagic kinase complexes ULK1 and VPS34 through CaMKKβ signaling (1). This mechanism is distinctly different from the TFEB-mediated slow-onset transcriptional regulation of the autophagy-lysosomal system, indicating that cellular responses to Ca^2+^ signals appearing in the vicinity of lysosomes span different time scales (1, 2). Supporting this argument, TRPML1 activity during prolonged starvation directs calmodulin to promote mTOR reassembly on the lysosome membrane, thereby providing a necessary brake on TFEB hyperactivation and autophagy (10). Finally, several studies have implicated potential roles for TRPML1 in lysosomal acidification. A steady H^+^ leak function of TRPML1 was proposed to prevent luminal over-acidification because TRPML1-deficient tissues progressively accumulate hyperacidic autolysosomes (11, 12). Conversely, 24-h TRPML1 activation by synthetic agonists in cell culture also leads to increased lysosomal acidification (13). Despite TRPML1’s importance as a regulator of lysosomal function, little is known about its rapid-onset responses, especially considering that agonist-induced TRPML1 Ca^2+^ signals have a duration of less than 30 s (4).

In this study, we identified a rapid (10–20 min) lysosomal activation response that is triggered by the TRPML1 agonist mucolipin selective agonist 1 (ML-SA1), which involves lysosomal acidification and increased lysosomal cathepsin activity. As an underlying mechanism, we show that early TRPML1-mediated Ca^2+^ efflux leads to local vesicle fusion events, which does not involve lysosomal redistribution to the perinuclear region that was reported earlier (1, 9). We show that this is achieved at least in part by TRPML1 promoting recruitment of the soluble N-ethylmaleimide–sensitive factor activating protein receptor (SNAREs) syntaxin 7 (Stx7) and vesicle-associated membrane protein 7 (VAMP7) to lysosomes, increasing vesicle fusion competence this way. In phosphatidylinositol 3,5-bisphosphate (PI(3,5)P_2_)-depleted cells (which is the sole known cellular agonist of this cation channel), Stx7 and VAMP7 are abnormally sequestered in the lysosome lumen. Importantly, pharmacological TRPML1 activation promotes Stx7 recycling to the plasma membrane in a process that is dependent on calmodulin. Taken together, we show that lysosomal maturation and activation responses immediately follow and rely on lysosomal calcium ion release *via* TRPML1.

## Results

### Acute TRPML1-mediated Ca^2+^ release induces lysosomal acidification and fusion responses

**Figure 1:**
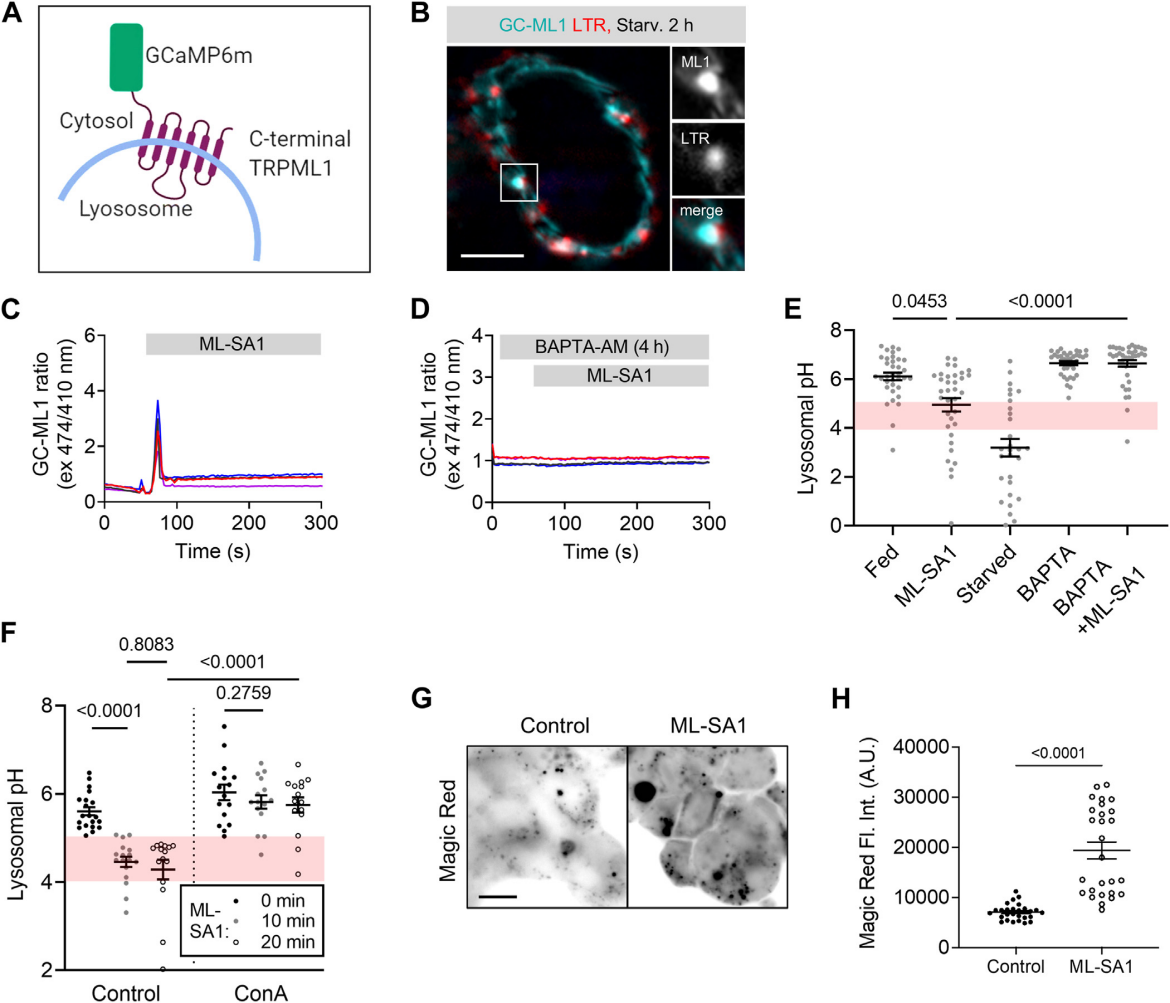
**Acute release of Ca**^**2+**^**by TRPML1 activates lysosomes.***A*, schematic of our GCaMP6m-TRPML1 (GC-ML1) lysosomal Ca^2+^ release sensor. *B*, upon amino acid starvation, GCaMP6 colocalizes with Lysotracker *Red* positive acidic vesicles (inset). Bar represents 5 μm. *C* and *D*, TRPML1-mediated Ca^2+^ release was recorded in GC-ML1 expressing HEK-293 control (*C*) and BAPTA-AM pretreated (2 μM, 4 h, *D*) cells after ML-SA1 induction (25 μM) using ratiometric live imaging (Ex 474 nm = Ca^2+^ sensitive, Ex 410 nm = Ca^2+^ independent) in a low external Ca^2+^ buffer (<10 nM free Ca^2+^). Ensemble total fluorescence measurements are shown from N = 4 experiments for both control and BAPTA-AM–treated conditions. *E*, ML-SA1 (25 μM, 15 min) induced acidification response in control cells stained with Lysosensor *Yellow*/*Blue*. Relative fluorescence ratio of *yellow* and *blue* emission channels were measured and compared against a standard curve to quantify lysosomal pH. *Red band* indicates lysosomal active pH range (4.0–5.0). BAPTA-AM pretreatment blocks the acidification response. Bar represents 10 μm. N = 37, fed; N = 36, ML-SA1; N = 29, Starved; N = 37, BAPTA; N = 39, BAPTA + ML-SA1. Statistics: Kruskal–Wallis one way ANOVA with Dunn’s *post hoc* test. *F*, lysosomal pH was measured based on FITC-dextran fluorescence quenching after 10 min and 20 min ML-SA1 treatment in control and concanamycin A (ConA; V-ATPase inhibitor, 100 nM, 1 h) pretreated cells, respectively. Optimal lysosomal pH range (4–5) is highlighted with *red* background. N = 15 to 20 for all samples. Statistics: two-way ANOVA with Tukey’s *post hoc* test. *G*, cathepsin B activity rapidly increases during a 15 min ML-SA1 treatment based on the conversion of its substrate *Magic Red*. Bar represents 5 μm. *H*, quantification of whole-cell *Magic Red* fluorescence. N = 27, statistics: two-tailed Mann-Whitney *u* test.

Multiple reports showed that TRPML1 activation promotes the degradation of neurotoxic cargo (14, 15). We decided to explore if TRPML1 regulates lysosomal acidification, since inactive lysosomes have a higher resting luminal pH (16). We generated a new biosensor (GCaMP6m-TRPML1, hereafter GC-ML1) to measure lysosomal Ca^2+^ release by TRPML1 (Fig. 1, *A* and *B*). This construct was used along with Lysosensor Yellow/Blue to follow Ca^2+^ release and lysosomal acidification responses in parallel. The commonly used synthetic TRPML1 agonist ML-SA1 elicited rapid Ca^2+^ release and increased lysosomal acidity as evidenced by elevated Lysosensor Yellow/Blue fluorescence ratio within 15 min of treatment, both of which were blocked by prior intracellular Ca^2+^ chelation by BAPTA-AM (Fig. 1, *C*–*E*). After 10 min of TRPML1 activation, lysosomal pH decreased to the level needed for optimal acidic hydrolase activity (pH = 4.45 after 10 min *versus* pH = 5.6 before treatment). We confirmed this using another ratiometric dye fluorescein isothiocyanate (FITC)-dextran that specifically localizes to lysosomes upon a defined pulse-chase period: we found that these TRPML1-activated lysosomes did not acidify further by 20 min of treatment (Fig. 1*F*), indicating that the acidification response is much faster than suggested by previous reports (13, 15). Inhibition of acidification by the vacuolar ATPase (v-ATPase) inhibitor concanamycin A (ConA, Fig. 1*F*) showed that this process requires active proton pumping into lysosomes. We could not use BAPTA-AM in these ratiometric pH measurements because it interfered with lysosomal delivery of FITC-dextran. The v-ATPase proton pump consists of two domains: a membrane-integral V_0_ and a peripherally associated V_1_. To test if TRPML1 increased v-ATPase assembly to promote acidification, we measured ATP6V1A levels on immunopurified lysosomes (Fig. S1*A*). Unlike starvation-induced acidification where lysosomal V1 localization is drastically increased, V1 levels only mildly altered during 20 min of ML-SA1–triggered acidification (Fig. S1*B*), which probably explains why starvation triggers more robust lysosome acidification than ML-SA1 treatment (Fig. 1*E*). Next, we measured V1 localization using low-level expression of GFP-SidK, a fluorescent reporter for V1A (17). While SidK puncta number only mildly increased during ML-SA1 treatment, LysoTracker (a commonly used staining for acidic lysosomes) positivity of SidK puncta increased drastically (Fig. S1, *C*–*E*). Finally, increased cathepsin B activity was observed in cells after 15 min of ML-SA1 treatment (Fig. 1, *G* and *H*). Taken together, these data indicate that TRPML1 Ca^2+^ release predominantly relies on preassembled v-ATPase complexes to promote lysosomal acidification and degradation.

**Figure 2:**
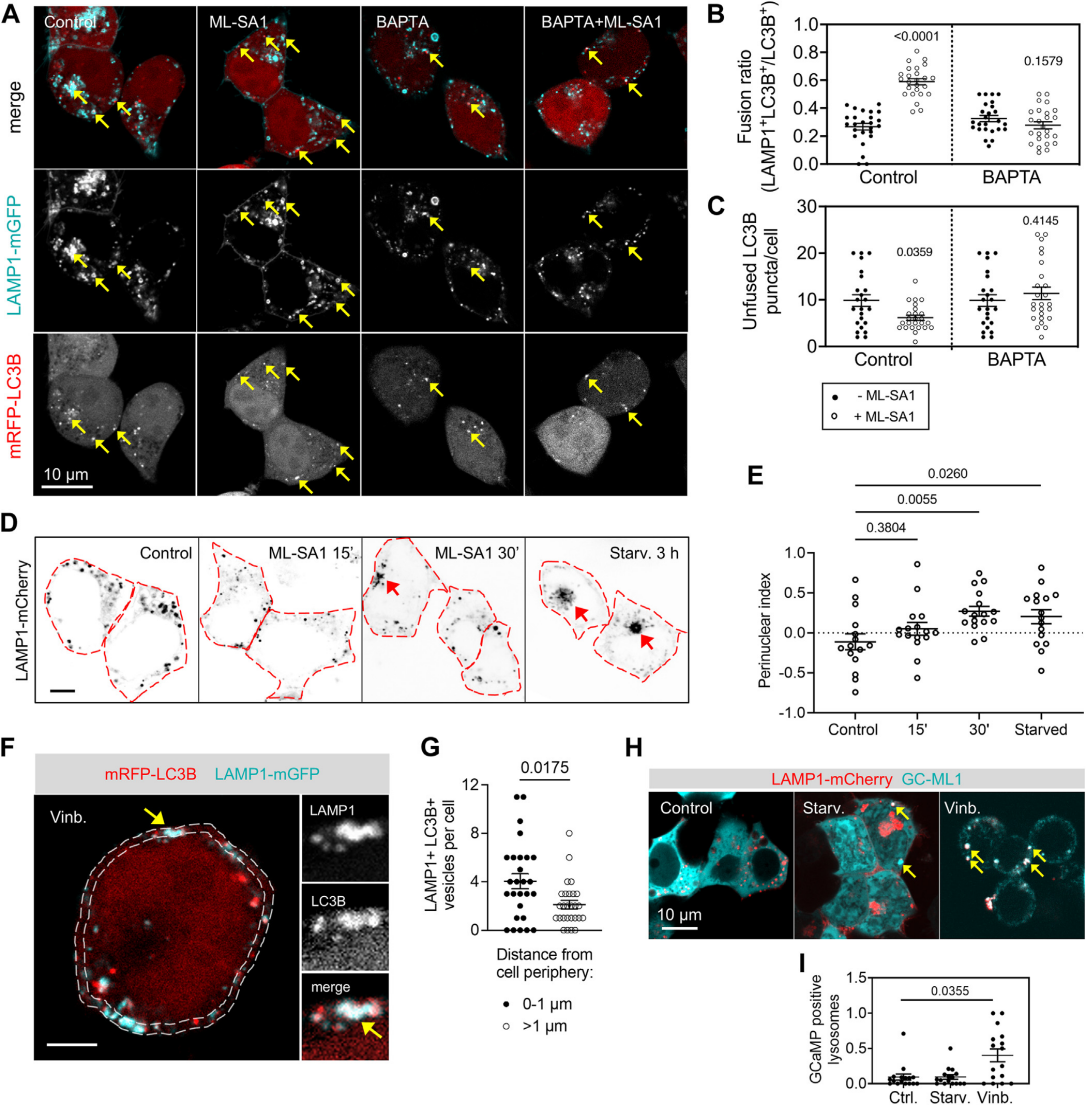
**Increased autophagosome-lysosome fusion at early stages of TRPML1 activation is independent of lysosome migration.***A*, autophagosome-lysosome fusion was assayed by LAMP1-mGFP and mRFP-LC3B colocalization in control or BAPTA-AM pretreated (2 μM, 4 h) cells in response to ML-SA1 induction (25 μM, 15 min). *Arrows* indicate colocalized puncta. Bar represents 5 μm. *B*, quantification of colocalization (fusion) ratios of LC3B-positive autophagic structures with LAMP1 (lysosomes). N = 25. Statistics: Mann-Whitney *u* test: Control and ML-SA1; two-tailed unpaired *t* test: BAPTA-AM and BAPTA-AM + ML-SA1. *C*, unfused (LAMP1-negative) LC3B puncta number decreases during short ML-SA1 treatment, suggesting that autophagosome biogenesis is not increased. N = 23, control; N = 25, ML-SA1; N = 23, BAPTA; N = 25, BAPTA + ML-SA1. Statistics: two-tailed Mann-Whitney *u* tests. *D*, lysosome localization in cells transfected with LAMP1-mCherry and treated with ML-SA1 for 15 min, 30 min, or starved in EBSS for 3 h. Bar represents 5 μm. *Red arrows* indicate lysosome clusters. *E*, perinuclear lysosome clustering was calculated as described in Experimental procedures from cells such as those shown in (*D*). N = 15, Control; N = 16, ML-SA1 (15′ and 30′), starved. Statistics: one way-ANOVA with Dunnett’s *post hoc* test. *F*, vinblastine-treated cell (20 μM, 2.5 h) is showing clear colocalization of LAMP1 (lysosomal) and LC3B (autophagosomal) signals (*arrow*). Cell periphery (0–1 μm) is highlighted with *dotted lines*. Bar represents 5 μm. *G*, vinblastine-treated cells show peripheral accumulation of autolysosomes. N = 27, statistics: two-tailed Mann-Whitney *u* test. *H*, vinblastine-treated cells contain more lysosomes that are GC-ML1-positive (*arrows*) than untreated and starved cells. Bar represents 5 μm. *I*, quantification of GC-ML1–positive LAMP1 structures in control, starved, and vinblastine-treated cells. N = 16, statistics: Kruskal–Wallis test with Dunn’s *post hoc* test.

Earlier studies showed that activation of TRPML1 promotes autophagosome biogenesis, culminating in increased autophagosome-lysosome fusion *via* lysosomal migration along microtubules (1, 9). However, the rapid, 10 to 20 min onset of lysosomal activation coupled to peri-lysosomal Ca^2+^ increase in our experiments raised the possibility that lysosomes could fuse more effectively with nearby autophagosomes during this period. Fifteen minutes of ML-SA1 treatment indeed increased colocalization between lysosomal-associated membrane protein 1 (LAMP1) and LC3B puncta, with a concomitant decrease of LAMP1-negative LC3B puncta (*i.e.* unfused autophagosomes), which was blocked by intracellular Ca^2+^ chelation (Fig. 2, *A*–*C*). We have also measured perinuclear lysosomal migration during this time, which was unchanged at 15 min while lysosomes started to cluster near the nucleus starting at around 30 min after ML-SA1 administration, resembling the already described perinuclear localization of lysosomes after 3 h starvation (Fig. 2, *D* and *E*) (9).

These results suggested that the majority of vesicle fusions that are attributed to ALG-2–mediated lysosomal migration upon prolonged TRPML1 agonist treatments lead to fusions with actively migrating (auto) lysosomes, while immediate fusion events with autophagosomes upon short treatments happen locally and do not necessarily involve vesicle transport along microtubules. In support of this, the majority of LAMP1+ vesicles still contained autophagosome-derived LC3B signal when the microtubule inhibitor vinblastine was applied to cells to block lysosomal migration, but their colocalization could be inhibited upon bafilomycin A1 cotreatment (Figs. 2, *F* and *G*, and S2*A*). Moreover, lysosomes in vinblastine-treated cells showed increased GC-ML1 fluorescence relative to control and starved cells (Fig. 2, *H* and *I*). This suggests that lysosome migration-independent local fusion is coupled to elevated TRPML1-mediated Ca^2+^ efflux. Vinblastine-treated cells also exhibited significant lysosomal accumulation of the TRPML1 agonist PI(3,5)P_2_ and contained significantly larger lysosomes (Fig. S2, *B*–*D*). This is in line with studies showing that PI(3,5)P_2_ synthesis is elevated during homotypic fusion between late endosomes/lysosomes (18, 19). This could explain the observed increase in TRPML1 activity and local autophagosome-lysosome fusions involving nonmigrating lysosomes. Overall, we conclude that pharmacological activation of TRPML1 is sufficient to rapidly promote local autophagosome-lysosome fusion and lysosomal acidification responses.

### Loss of TRPML1 function impairs lysosomal activity during starvation

**Figure 3:**
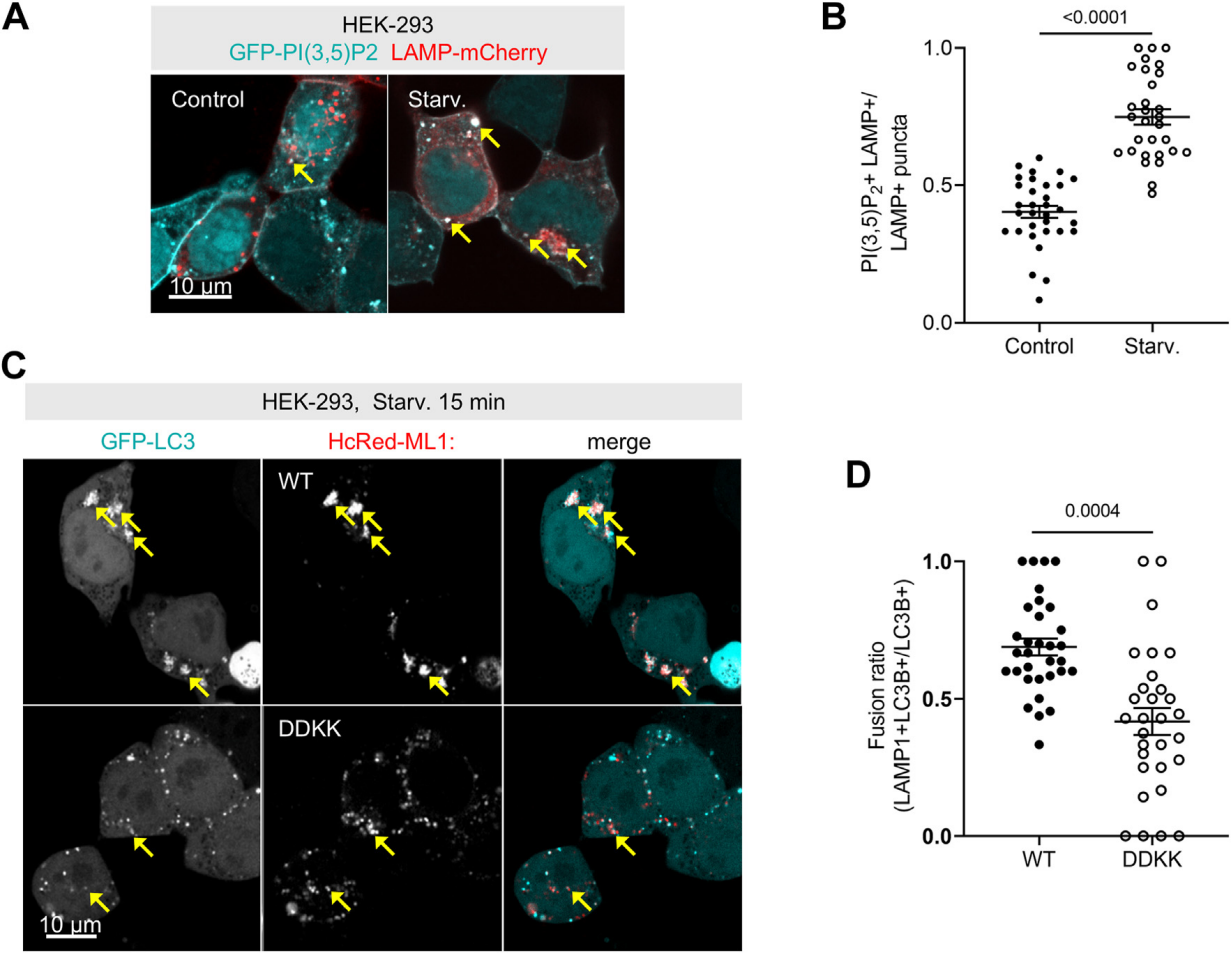
**Loss of TRPML1 activity during amino acid starvation impairs lysosomal fusion and acidification responses.***A*, HEK-293 cells expressing GFP-PI(3,5)P_2_ and LAMP-mCherry show marked increase in lysosomal PI(3,5)P_2_ upon starvation (*arrows*). Bar = 5 μm. *B*, quantification of PI(3,5)P_2_ presence on lysosomes. N = 32, control; N = 30, starvation. Statistics: two-tailed unpaired *t* test. *C*, HEK-293 cells were transfected with GFP-LC3B and HcRed-labeled TRPML1 WT or TRPML1 DDKK and starved in EBSS for 30 min. *Arrows* point to colocalized puncta, which are more frequent in TRPML1 WT cells. Bar represents 5 μm. *D*, percentage colocalization of HcRed (lysosomes) with GFP-LC3B puncta (autophagosomes) as a readout for autophagosome-lysosome fusion. N = 15, statistics: two-tailed unpaired *t* test.

Since TRPML1 currents are active during starvation, we wanted to see if its physiological agonist lysosomal PI(3,5)P_2_ is also upregulated. Indeed, 2 h starvation remarkably increased lysosomal PI(3,5)P_2_ levels (Fig. 3, *A* and *B*). We next overexpressed WT HcRed-TRPML1 WT or a mutant version: HcRed-TRPML1 DDKK (D471K/D472K), which interferes with the formation of the multimeric cation channels (20), to further test the role of TRPML1 during the starvation response. TRPML1 DDKK hetero-oligomerizes with endogenous TRPML1 so its overexpression acts in a dominant-negative manner in terms of ion conductance, and this partial loss-of-function potentially also avoids pleiotropic and indirect effects of a complete knockout (21). We first confirmed that TRPML1 DDKK expression indeed attenuated the rise of intracellular Ca^2+^ levels compared to TRPML1 WT expression when ML-SA1 was administered (Fig. 3, *A* and *B*). We also observed much less Lysosensor green fluorescence in TRPML1 DDKK than in TRPML1 WT overexpressing cells, indicating that the dominant-negative mutant protein indeed impaired lysosomal acidification (Fig. 3, *C* and *D*). In terms of autophagosomal fusion, TRPML1 DDKK–associated lysosomes showed lower colocalization with LC3B+ autophagosomal cargo than in case of TRPML1 WT, and TRPML1 WT positive lysosomes were also prominently clustered as opposed to TRPML1 mutant ones (Fig. 3, *C* and *D*), in line with TRPML1’s role in promoting lysosomal clustering (9). These results support that lysosomal acidification and fusion events occur early on in response to TRPML1 activity and show that this is relevant during the starvation response.

### Autophagosome fusion promotes TRPML1-mediated lysosomal Ca^2+^ efflux and acidification

To directly compare TRPML1 activity of autolysosomes (that is, those that have already received autophagic cargo) *versus* naïve lysosomes, we measured their ML-SA1–induced maximal GC-ML1 response (F_max_-F_0_/F_0_) after starvation. We did not see major differences in this (Fig. 3, *E* and *F*), indicating that lysosomal Ca^2+^ is not depleted during fusions. We did not see any differences in the GC-ML1 response of lysosomes based on their cellular distribution either, although their basal TRPML1 activity seemed to correlate with their proximity to the nucleus and presumably the perinuclear ER network, the primary source of organellar Ca^2+^ that drives Ca^2+^ refilling of lysosomes after lysosomal Ca^2+^ efflux (Fig. S3, *G*–*I*).

**Figure 4:**
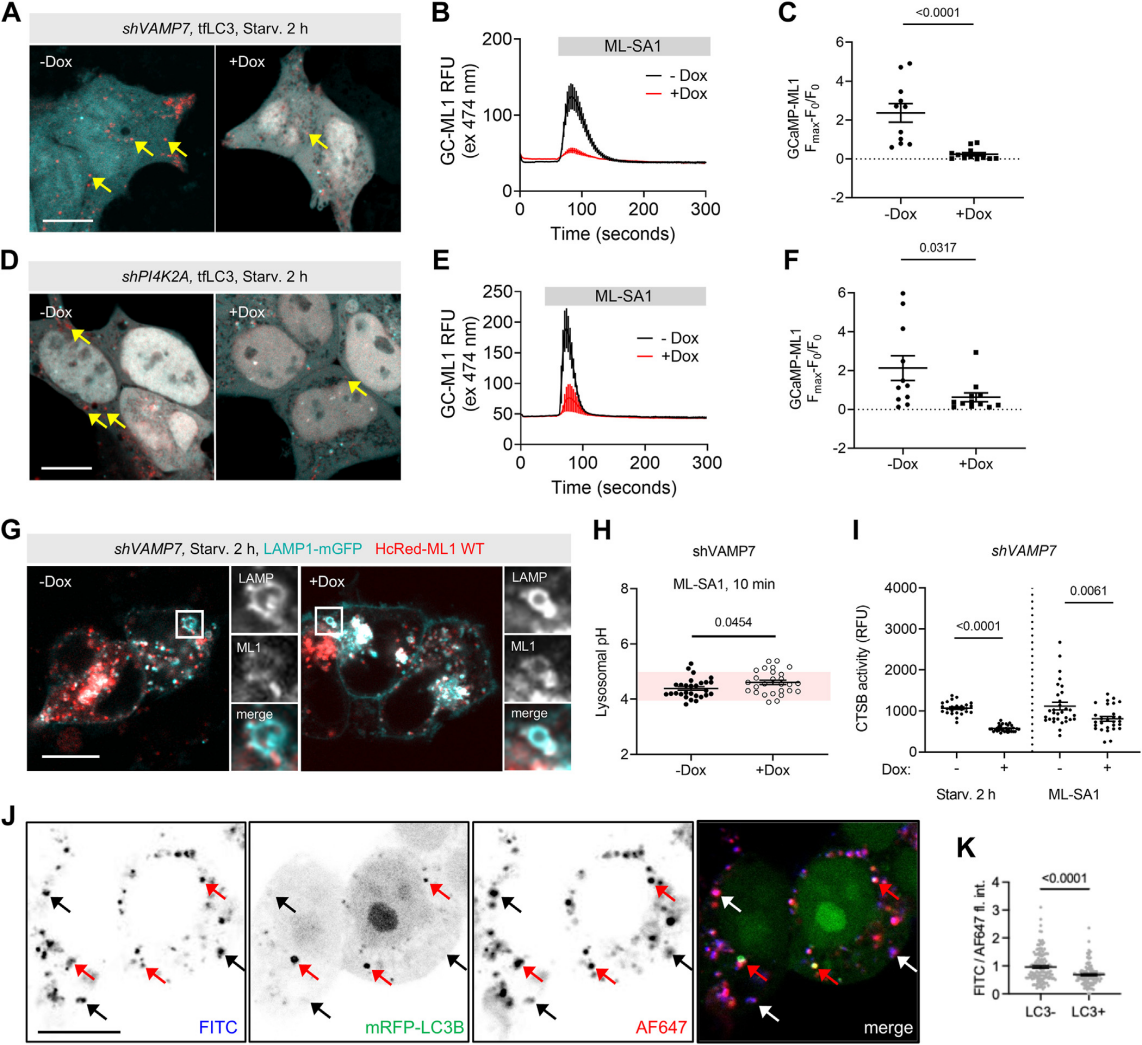
**TRPML1-mediated lysosomal Ca**^**2+**^**efflux and acidification responses require lysosomal fusions.***A*, stable shVAMP7 cells were incubated with 2 μg/ml Dox for 72 h to induce knockdown. Autophagosome-lysosome fusion was assayed in shVAMP7 cells by mRFP-EGFP-LC3B (“tfLC3” for tandem fluorescent LC3B) after 2 h starvation in EBSS. *Arrows* indicate autolysosomes, that is, fused autophagosomes (*red dots*), which lost GFP fluorescence due to its quenching in the acidic lysosomal lumen. *B*, GC-ML1 response to ML-SA1 was attenuated by VAMP7 knockdown. N = 11, −Dox; N = 12, +Dox. *C*, quantification of GC-ML1 response in shVAMP7 cells. Statistics: two-tailed Mann-Whitney *u* test. *D*, stable shPI4K2A-expressing cells were incubated with Dox to induce knockdown. Autophagosome-lysosome fusion was assayed by tfLC3 as in (*A*). *E*, GC-ML1 response to ML-SA1 was attenuated in shPI4K2A. N = 6. *F*, quantification of GC-ML1 response in shPI4K2A cells. Statistics: two-tailed Mann-Whitney *u* test. *G*, localization of TRPML1 on the lysosomal membrane was unchanged in shVAMP7 fusion-deficient cells (inset highlights membrane localization). Bar represents 5 μm. *H*, lysosomal acidification was quantified based on lysosomal FITC-Dx quenching in shVAMP7 cells during ML-SA1 treatment, revealing a statistically significant impairment in knockdown cells. N = 28, −Dox; N = 27, +Dox. Statistics: two-tailed unpaired *t* test. *I*, cathepsin B activity was measured by *Magic Red* staining in shVAMP7 cells during starvation (EBSS, 2 h) and ML-SA1 treatment (25 μM, 20 min). Relative whole-cell *Magic Red* fluorescence values were quantified from all samples imaged under the same experimental conditions. N = 25, -Dox (starved); N = 31, +Dox (starved); N = 31, −Dox (ML-SA1), N = 28, +Dox (ML-SA1). Data was analyzed by two-tailed Mann-Whitney *u* tests for starvation and ML-SA1, respectively. *J*, U2OS cells were transfected with mRFP-LC3B and pulsed overnight with FITC-Dx and Alexa Fluor 647 (used as a pH insensitive control tracer), followed by a 15 min chase period in EBSS to initiate starvation. LC3B associations with FITC-positive structures were counted as autolysosomes (*red arrow*), while LC3B-negative FITC structures were counted as unfused lysosomes (*black*/*white arrows*). FITC quenching, which is proportional to luminal acidification, is more pronounced in LC3B+ FITC + structures (autolysosomes). Bar represents 5 μm. *K*, FITC/AF647 fluorescence of individual LC3B+ and LC3B− structures were quantified for single vesicles. (N = 139, LC3−; N = 110, LC3+). Statistics: two-tailed Mann-Whitney *u* test.

To investigate the requirement of lysosomal fusions for TRPML1-induced acidification, we employed two strategies. First, we knocked down VAMP7, a lysosomal SNARE involved in autophagosomal and endosomal fusions. Second, we knocked down phosphatidylinositol 4-kinase type 2-alpha (PI4K2A), which synthesizes the fusion-critical lipid PI(4)P on autophagosomes (22) that recruits the autophagosomal SNARE protein STX17 (23, 24). We confirmed the efficiency of both knockdowns (Fig. 4, *A* and *B*). To exclude the possibility that lysosomal damage occurs in our cells, we confirmed that neither short-term ML-SA1 treatment nor these transient knockdowns affect lysosomal membrane integrity based on lack of galectin-3 recruitment to lysosomes, using a known lysosomal damage inducer as positive control (Fig. S4, *C*–*F*). We also used a tandem fluorescent GFP-mRFP-LC3B reporter (tfLC3) whose GFP fluorescence is quenched but mRFP fluorescence is retained in the acidic lysosomal environment. Lysosomal membrane lipidation is a hallmark of lysosomal damage (25) where lipidated LC3 decorates only the cytosolic side of the lysosomal membrane, therefore GFP quenching does not occur. The appearance of many red only (GFP- mRFP+) dots upon ML-SA1 treatment in different cell types (Fig. S4*G*) indicates that the source of lysosomal LC3 signals is the lysosomal delivery of autophagic cargo and not membrane lipidation. Knocking down *PI4K2A* or *VAMP7* interfered with autophagosome-lysosome fusion, as measured by tfLC3 (Figs. 4, *A* and *D*, and S4*H*). Importantly, both knockdowns also prevented proper Ca^2+^ release from lysosomes, indicated by attenuated ML-SA1–induced GC-ML1 response (Fig. 4, *B*, *C*, *E*, and *F*).

To further delve into the short-term effect of vesicle fusion on lysosomal activation, we chose to focus on VAMP7 because it is a lysosomal SNARE implicated in both endosome-lysosome and autophagosome-lysosome fusions (26). VAMP7 knockdown cells showed the expected localization of TRPML1 on LAMP1-positive vesicles, indicating that TRPML1 distribution was unaffected (Fig. 4*G*). Importantly, VAMP7 knockdown cells showed a statistically significant acidification defect upon pharmacological TRPML1 activation, along with a concomitant defect in cathepsin activity both during starvation and ML-SA1–induced TRPML1 activation (Fig. 4, *H* and *I*). We next followed acidification of individual lysosomes containing or lacking autophagic cargo (LC3B+ *vs*. LC3B−) during a short starvation period (15 min). In this experiment, Alexa Fluor 647-dextran was used as a nonreactive control to exclude potential dilution of FITC within lysosomes upon autophagic fusions. FITC quenching (FITC/AF647 ratio) that reflects acidification was indeed less efficient in LC3B− lysosomes, indicating that autophagosome-lysosome fusion induces lysosome acidification (Fig. 4, *J* and *K*). Taken together, we conclude that lysosomal fusion with incoming cargo-transporting vesicles triggers Ca^2+^ release, promotes acidification, and enhances hydrolytic enzyme activity of lysosomes for proper cargo breakdown.

### TRPML1 activation promotes SNARE delivery to lysosomes

**Figure 5:**
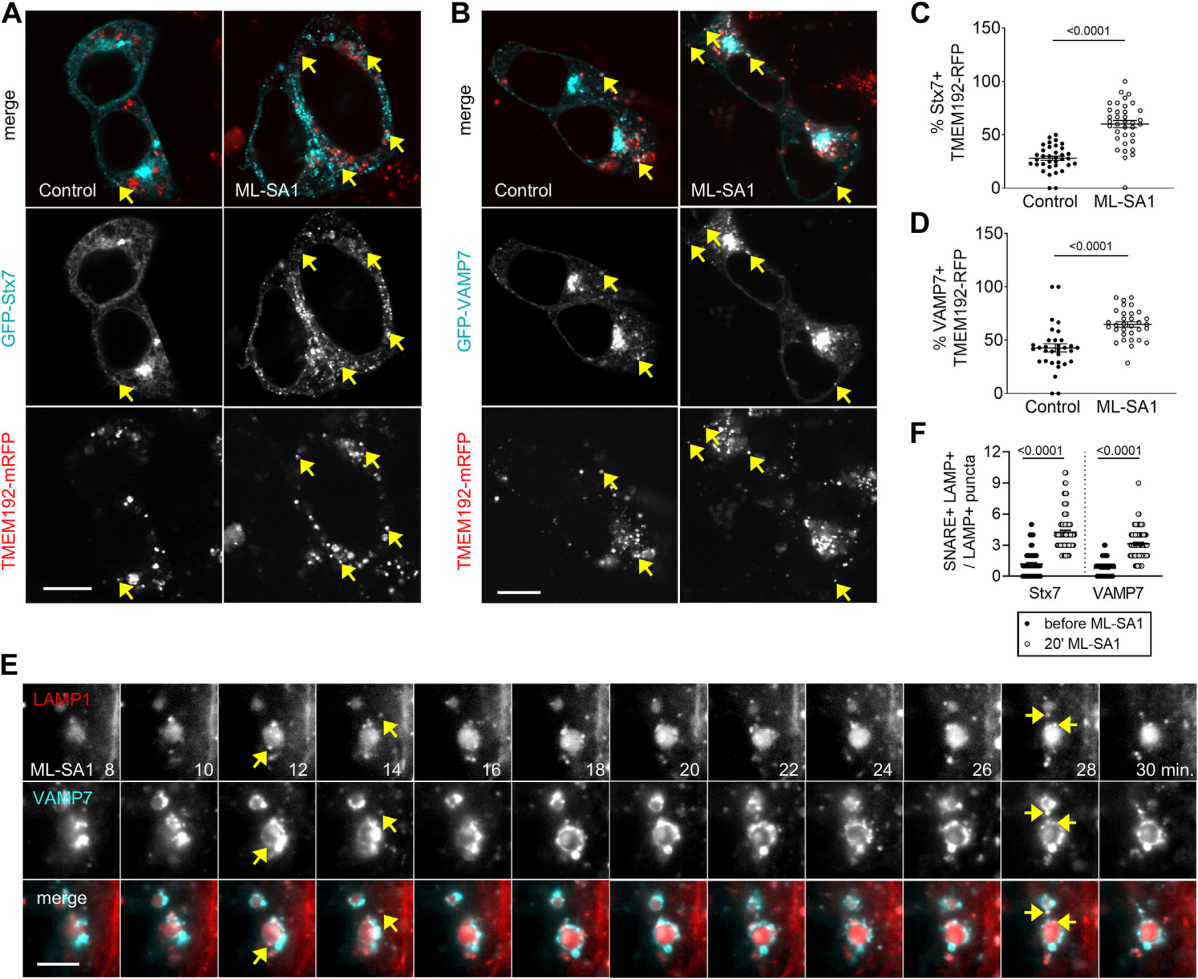
**Lysosomal enrichment of the Qa-SNARE Stx7 and R-SNARE VAMP7 following TRPML1-mediated Ca**^**2+**^**efflux from lysosomes.***A* and *B*, increased lysosomal localization of GFP-Stx7 (*A*) and GFP-VAMP7 (*B*) in ML-SA1–treated cells. *Arrows* point to colocalizing dots. Bars represents 5 μm. *C* and *D*, quantification of Stx7 (*C*) and VAMP7 (*D*) localization on TMEM192-positive vesicles (lysosomes). N = 35; statistics: unpaired two-tailed unpaired *t* test (Stx7), N = 32; statistics: two-tailed Mann-Whitney *u* tests (VAMP7). *E*, live imaging of U2OS cells expressing mCherry-LAMP1 and GFP-VAMP7 during the indicated periods after ML-SA1 treatment. Smaller carrier vesicle association near the periphery of the larger LAMP1-positive structure (lysosome) in the center is observed here 12 min onwards of ML-SA1 treatment, eventually leading to VAMP7 appearing at the lysosome membrane. *Yellow arrows* point to carrier vesicles positive for both LAMP1 and VAMP7. LAMP1 enrichment of the lysosome is also visible. Bar represents 5 μm. *F*, the number of SNARE-positive puncta at the rim of LAMP1-positive vesicles were counted before and after 20 min of ML-SA1 treatment. N in100 for all samples. Statistics: two-tailed Mann-Whitney *u* test.

We wondered whether the recruitment of VAMP7 and more generally, lysosomal SNAREs could increase following TRPML1 activation to facilitate lysosomal fusions. Both Stx7 and VAMP7 have well-defined roles in autophagosome-lysosome fusion: the autophagosomal SNARE Stx17 forms a SNARE complex with SNAP29 and VAMP7/VAMP8 to promote fusion (26), and Stx7 forms an alternative complex with SNAP29 and Ykt6 (27). VAMP7 is also part of a SNARE complex with Stx7 and Vti1b functioning at late endosome-lysosome membrane fusions (28, 29). We observed increased localization of VAMP7 and Stx7 on lysosomes after ML-SA1 treatment (Fig. 5, *A*–*D*). The increased localization of these SNAREs to lysosomes raised the possibility that TRPML1 promotes rapid lysosomal maturation to increase fusion competence.

A previous study reported that VAMP7 and vacuolar protein sorting–associated protein 41 homolog (Vps41) are transported by Golgi-derived LAMP1-positive carrier vesicles to lysosomes, a pathway parallel to the clathrin-mediated transport of lysosomal enzymes (30). We hypothesized that acute TRPML1-mediated Ca^2+^ release could facilitate the clustering and fusion of SNARE-containing carrier vesicles to lysosomes. These vesicles are typically concentrated around late endosomes/lysosomes (30). Another clue for this was that LAMP1 carriers preferentially fuse with mature late endosomes, while clathrin-coated carriers fuse with early endosomes. Thus, we live-imaged LAMP1 together with Stx7 or VAMP7 during ML-SA1 treatment. We indeed observed the association of smaller VAMP7- and LAMP1-positive vesicles next to larger LAMP1 structures (possibly late endosomes/lysosomes) starting at 10 min of ML-SA1 treatment and subsequent enrichment of VAMP7 signal in larger LAMP1 structures (Fig. 5, *E*, *arrows* and *F*). Stx7 trafficking followed similar dynamics (Figs. 5*F* and S5*A*). This result suggests that TRPML1 promotes lysosomal fusion competence by SNARE recruitment *via* LAMP1 carriers.

### Inhibition of PI(3,5)P_2_ synthesis perturbs lysosomal SNARE distribution

**Figure 6:**
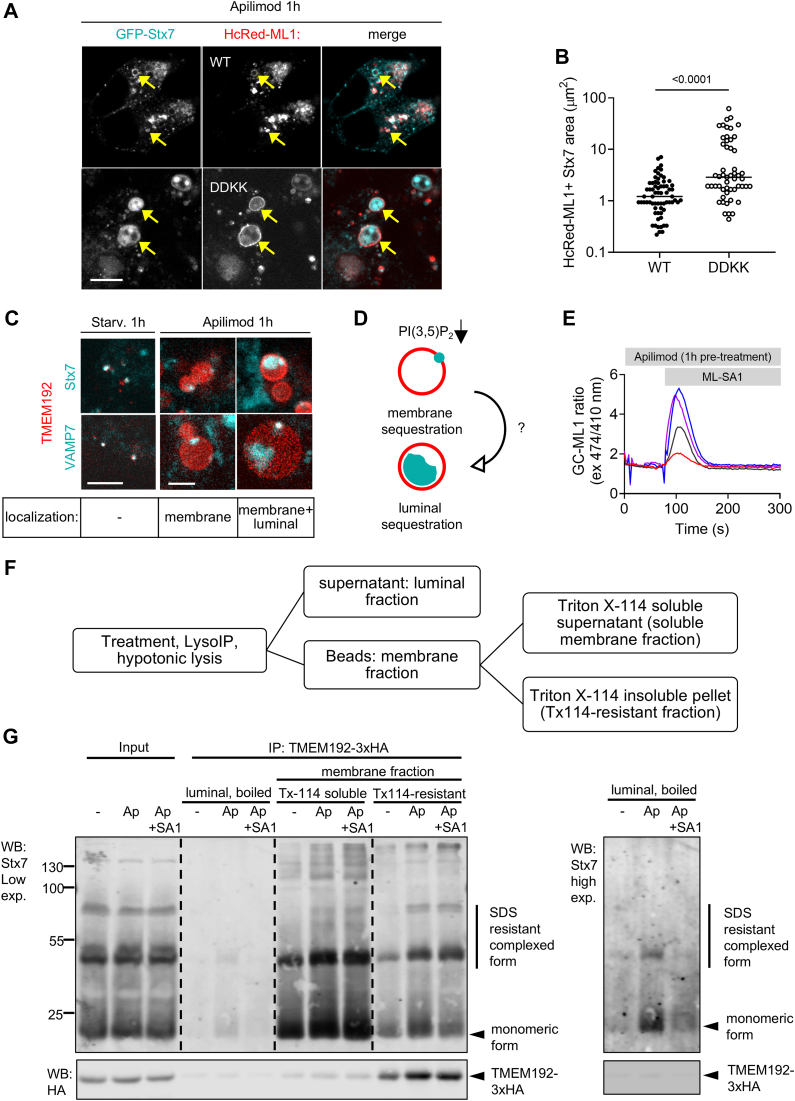
**TRPML1 activity rescues abnormal lysosomal SNARE distribution in PI(3,5)P**_**2**_**-deficient cells.***A*, HEK-293 cells were transfected with GFP-Stx7 and HcRed-fused TRPML1 WT or TRPML1 DDKK and then treated with Apilimod (Ap; 1 h) to inhibit PI(3,5)P_2_ synthesis by PIKfyve. *Arrows* indicate TRPML1-positive structures (lysosomes) containing Stx7 signal. Bar represents 5 μm. *B*, significant increase in the size of Stx7+ TRPML1+ structures were seen in Ap-treated TRPML1 DDKK cells, indicating more Stx7 sequestration. N = 68, wt; N = 53, DDKK. Data was analyzed using two-tailed Mann-Whitney *u* test. *C*, individual lysosomes labeled by TMEM192-mRFP and co-labeled with lysosomal SNAREs (either Stx7 or VAMP7) during starvation (EBSS 1 h) and Ap-treated conditions. In the absence of PI(3,5)P_2_ synthesis, SNAREs are present in membrane-associated puncta and also sequestered inside the lumen. Bars represent 5 μm. *D*, cartoon depicting typical SNARE localization in PIKfyve-inhibited cells, showing foci-like distribution on the membrane (*top*), as well as luminal sequestration (*bottom*) possibly at a more advanced stage. *E*, ML-SA1 induction of GC-ML1 response in Ap-treated cells; N = 4. *F*, scheme of sample preparation for the fractionation of lysosomal proteins from TMEM192-3xHA–expressing cells into membrane (Triton X-114 soluble and resistant) and luminal fractions. *G*, distribution of endogenous Stx7 in monomeric and an SDS-resistant, complexed form increased in the Triton X-114 resistant fraction upon Ap treatment, as well as the amount of luminal Stx7 was increased (*cf.* high-exposure blot on the *right*). Co-incubation of Ap with ML-SA1 strongly reduced the amount of sequestered luminal Stx7 as well as moderately reduced monomeric Stx7 in the Triton X-114–resistant membrane fraction (which also contains the lysosomal integral membrane protein TMEM192), both indicating restored SNARE recycling from the lysosome.

PI(3,5)P_2_ is a minor but key signaling phospholipid in the lysosome membrane, synthesized exclusively by PIKfyve in mammals. PIKfyve-deficient cells show impaired autophagosome-lysosome fusion and enlarged, nondegradative lysosomes (31, 32, 33). Notably, PI(3,5)P_2_ is the only known cellular agonist of TRPML1, and interestingly, degradative defects of PIKfyve-inhibited lysosomes can be rescued by ML-SA1 (31). Moreover, TRPML1-released Ca^2+^ and calmodulin activation can rescue the lysosome enlargement or ‘coalescence’ phenotype observed due to PIKfyve inhibition (34). We wanted to investigate the role of PI(3,5)P_2_ in the context of its agonist function on TRPML1. One hour treatment with the PIKfyve inhibitor apilimod impaired PI(3,5)P_2_ synthesis and recapitulated the published lysosome coalescence phenotypes (Fig. S5, *B*–*D*). We wondered whether SNARE mislocalization could contribute to the fusion defect during PIKfyve inhibition, given that SNAREs are known to be sequestered on the lysosome membrane in lysosomal storage disorders that alter cholesterol metabolism (35). During apilimod-mediated lysosomal enlargement, membrane association of Stx7 became obvious in TRPML1 WT lysosomes, while large Stx7 deposits were seen inside lysosomes in TRPML1 DDKK (Fig. 6, *A*, *yellow arrows* and *B*). Stx7 and VAMP7 were also clearly present on lysosomes in starved cells but their membrane association was not clear, given that these vesicles are relatively small (Fig. 6*C*, *left*). Apilimod treatment also led to highly aberrant Stx7 and VAMP7 localization even without TRPML1 overexpression (Fig. 6*C*), with these SNAREs appearing either as distinct foci on the lysosome membrane (Fig. 6*C*, *middle panel*) or sequestered within the lysosome lumen (Fig. 6*C*, *right panel*). This may reflect distinct, progressive stages of aberrant lysosomal SNARE sequestration (Fig. 6*D*).

### SNARE distribution and autophagosome-lysosome fusion is restored by the activation of TRPML1 in cells lacking lysosomal PI(3,5)P_2_

PI(3,5)P_2_ and ML-SA1 can synergistically activate TRPML1 owing to distinct, allosteric binding sites (36); thus ML-SA1 can trigger the release of lysosomal Ca^2+^ in apilimod-treated cells. Indeed, we found that the GC-ML1 response in apilimod-treated cells was somewhat delayed and more heterogeneous but ultimately comparable to untreated control cells (F_max_ = 3.82 *versus* 2.31, Fig. 6*E*, see also Fig. 1*C*). ML-SA1 also restored normal localization of Stx7 and VAMP7 in apilimod-treated cells (Fig. S5, *D*–*G*). This is in line with the differences in Stx7 distribution between apilimod-treated TRPML1 WT and TRPML1 DDKK lysosomes (Fig. 6, *A* and *B*), as described above.

**Figure 7:**
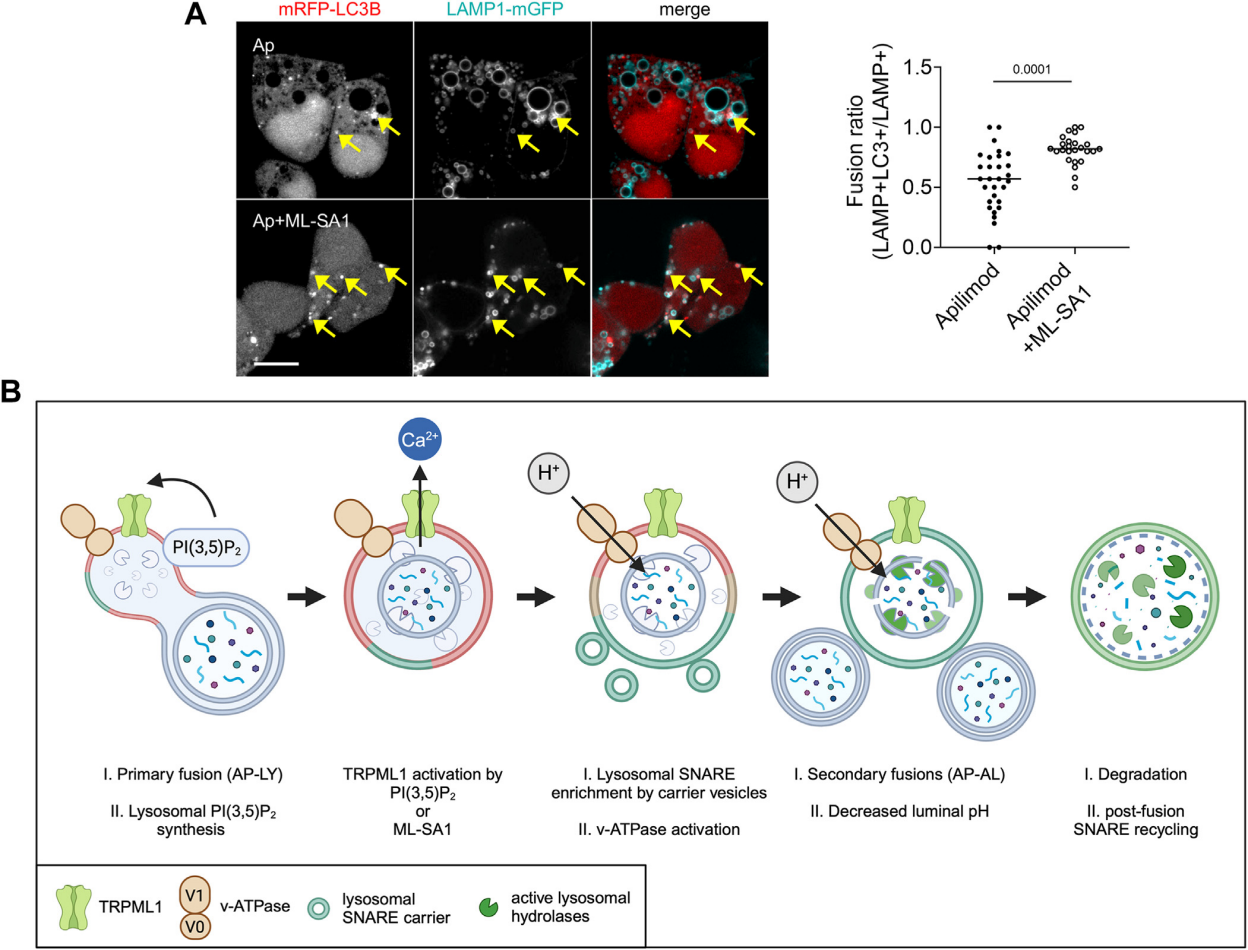
**TRPML1 activation restores lysosomal fusion in PI(3,5)P**_**2**_**-deficient cells.***A*, cells were transfected with the indicated constructs and treated with apilimod (Ap, 1 h), which impairs autophagosome-lysosome fusion. ML-SA1 cotreatment rescued the fusion activity of these lysosomes (*arrows* indicate fused autophagosomes). Bar represents 5 μm. *Right*: quantification of the results shows significantly increased fusion rate. N = 30, Ap; N = 25, Ap+ML-SA1. Statistics: two-tailed unpaired *t* test. *B*, schematic representation of the role of TRPML1 in regulating lysosomal degradative function during autophagy. The first autophagosome-lysosome fusion(s) (AP-LY) coupled to lysosomal PI(3,5)P_2_ synthesis activates TRPML1. Upon TRPML1 activation, lysosomal acidification and recruitment of SNAREs Stx7 and VAMP7 *via* SNARE carrier vesicles are increased, followed by secondary fusions of these autolysosomes with autophagosomes (AP-AL) and further acidification. Created with Biorender.com.

To biochemically verify this finding, we resorted to LysoIP-based purification and fractionation of lysosomal proteins into luminal, Triton X-114 soluble and insoluble membrane fractions from control, apilimod only, and apilimod + ML-SA1–treated cells (Fig. 6*F*). Corroborating a previous report in cells derived from a lysosomal storage disorder mouse model (35), we saw increased formation of likely nonfunctional Stx7 supercomplexes upon apilimod treatment in the TX-114 insoluble fraction (Fig. 6*G*), indicating its sequestration on complex, perhaps cholesterol-rich membrane compartments akin to lipid rafts. Moreover, Stx7 accumulation was visible mostly in the luminal fraction in both monomeric and complexed forms, which were significantly reduced upon ML-SA1 cotreatment (Fig. 6*G*, *right panel*). The amount of monomeric, presumably active form of Stx7 and its complexed forms associated with membranes remained unchanged upon ML-SA1 cotreatment. Together with the microscopy data, these indicate that TRPML1 activity prevents the luminal sequestration of Stx7 during PIKfyve inhibition, possibly also facilitating the recycling of monomeric Stx7 from the lysosome membrane. In line with this, 15-min ML-SA1 treatment of apilimod pretreated cells restored autophagosome-lysosome fusion (Fig. 7*A*).

### Stx7 recycling in PI(3,5)P_2_-deficient lysosomes requires calmodulin

Postfusion recycling of lysosomal SNAREs including VAMP7 and Stx7 to the cell surface is regulated by dedicated clathrin adaptors and has been shown to depend on Ca^2+^/calmodulin (37, 38). Moreover, abnormal endolysosome size in PIKfyve-inhibited cells is restored upon TRPML1 activation, which is also a calmodulin-regulated process (34, 39). We therefore asked whether calmodulin is important for SNARE recycling in PIKfyve-inhibited cells. To address this, apilimod withdrawal and subsequent ML-SA1 treatment was combined with the calmodulin inhibitor W7. In cells with calmodulin inhibition, even ML-SA1 failed to increase peripheral Stx7 localization (Fig. S6). This indicates that the lysosomal SNARE recycling function of TRPML1 relies on calmodulin.

## Discussion

Here we have identified a direct role of TRPML1 channel opening in lysosomal maturation, based on increased fusion competence by lysosomal SNARE recruitment, more acidic lysosomal pH, and a concomitantly enhanced cathepsin hydrolase activity. Our results suggest that these functions are coupled and that lysosomal fusion promotes the activation of v-ATPase. In support of this, we show that fusion-deficient (VAMP7 knockdown) lysosomes fail to release this cation in response to ML-SA1 and that autolysosomes have faster acidification kinetics than naïve lysosomes during amino acid starvation. This is in line with our earlier studies showing that fusion-deficient or autophagy-deficient cells have lysosomes that remain lysotracker negative (*i.e.* nonacidic, thus proteolytically inactive) in starved *Drosophila* larval fat tissue (40, 41, 42). Our results also agree with an earlier study showing that starved *ATG5* KO cells fail to induce lysosomal hydrolytic activity (43). The last study did not measure the extent of lysosome damage in ATG5 KO cells, though. ATG5 is a key regulator of LC3 lipidation machinery and therefore it is activated upon lysosome damage (44). Our results firmly establish that undamaged lysosomes remain proteolytically inactive in the absence of incoming vesicular cargo. Strikingly, ML-SA1–elicited TRPML1 responses remained unchanged irrespective of cellular lysosomal distribution (perinuclear *versus* peripheral) as well as its autophagic fusion status, indicating a similar capacity of TRPML1 to be activated by its agonist in naïve and autolysosomes. Thus, differences in the pH of LC3B+ *versus* LC3B− lysosomes are likely due to the altered Ca^2+^ efflux kinetics post-fusion. The attenuation of TRPML1-mediated Ca^2+^ efflux in fusion-impaired cells demonstrates a critical role of lysosomal Ca^2+^ in the regulation of acidic pH.

The question arises: how does the fusion of incoming vesicles such as autophagosomes activate lysosomes? Two aspects must be considered here: first, PI(3,5)P_2_ is locally synthesized on lysosomes during later stages of autophagy, likely post-fusion (31, 45, 46) and thus it can act as the physiological agonist to activate TRPML1 during/after fusion. This is supported by the observation that autolysosomes containing undegraded cargo in our vinblastine-treated cells accumulated PI(3,5)P_2_. Second, lysosomal acidification during starvation relies on the reversible assembly of the v-ATPase holocomplex (comprising of cytosolic V1 and membrane-tethered V0 subunits), concomitant with the end-directed migration of lysosomes along microtubules (9, 17, 47). However, lysosomal V1 localization is not significantly altered within 20 min of ML-SA1 treatment (Fig. S1), indicating that TRPML1-regulated acidification relies mostly on already available preassembled holocomplexes. Since cytosolic Ca^2+^ chelation prevents this induced acidification, it depends on TRPML1-released Ca^2+^ and not due to ionic imbalances occurring in the lysosomal lumen that can also arise from TRMPL1 activation (48). It is tempting to speculate that the autophagosomal fusion itself activates the v-ATPase, which is also supported by a recent report about the binding between the N-terminal cytosolic domain of several V_0_ a subunits to specific phospholipids such as PI(3,5)P_2_ and PI(4)P for their activation (49). Among these, PI(3,5)P_2_ is synthesized on the lysosome membrane during autophagy (see above), and while PI(4)P is primarily abundant in Golgi, it is also synthesized during autophagosome maturation and required for their fusion with lysosomes (22, 23, 24). Further evidence is needed to demonstrate such signaling.

The failure to induce TRPML1-dependent Ca^2+^ release in VAMP7-deficient cells, for example, is not due to the mislocalization of TRPML1 itself. Instead, this could be due to a general defect in incoming vesicle fusions. The rapid recruitment of lysosomal SNAREs indicates a critical role of lysosomal Ca^2+^ efflux towards their functional maturation. This relies on the availability of SNARE carriers for Ca^2+^-dependent fusion, which is in turn regulated by VAMP7 and the HOPS vesicle tether subunit Vps41 (30, 50). It is possible that both carrier vesicle-lysosome fusions and autophagosome-lysosome fusions driven by lysosomal Ca^2+^ happen in parallel or at the same time, because: (1) VAMP7/VAMP8 KO cells accumulate both autophagic and LAMP carrier vesicles, (2) both fusions utilize the HOPS tethering complex (40, 51, 52). Interestingly, the knockout of VAMP7 and VAMP8 did not alter cathepsin delivery to lysosomes (52). This is in line with our observations and indicates that carrier vesicle fusion and lysosomal activation are distinctly different downstream responses of TRPML1 activation. These likely mediate: (1) biosynthetic delivery of cathepsins to lysosomes and (2) availability of carrier vesicles to enhance fusion competence, respectively.

The lysosomal enlargement/coalescence resulting from PIKfyve inhibition is reversible, and it is thought to be due to the inhibition of the ‘run’ or fission step of the constant ‘kiss-and-run’ lysosomal fusion-fission cycle that maintains lysosomal size (53, 54). A previous study indicated the presence of Magic Red (cathepsin activity marker) and Lysotracker Red fluorescence in distinct subcompartments of multi-lamellar PIKfyve-inhibited lysosomes (55). It is tempting to speculate that these subcompartments can sequester lysosomal SNAREs and impair their recycling by preventing the formation of clathrin-coated buds (35). We show that normal Stx7 recycling to the cell periphery is restored by lysosomal Ca^2+^ release in PIKfyve-inhibited cells that sequester Stx7 in large clumps within lysosomes. The “rescue” effect of lysosomal Ca^2+^ efflux is probably because lysosomal fission in PIKfyve-inhibited cells is directly promoted by the TRPML1 Ca^2+^-calmodulin axis, thus restoring correct vesicle size and membrane SNARE distribution (34).

Multiple studies established that TRPML1 activation facilitates cargo degradation, for example *via* inducing formation of new autophagosomes and lysosomes as well as their perinuclear transport (13, 14, 15, 22, 39). Here we identify a new mechanism by showing that incoming vesicle fusions rapidly activate lysosomes in a TRMPL1- and Ca^2+^-dependent manner, which then leads to increased local fusion events between autophagosomes and lysosomes to speed up autophagic cargo degradation (Fig. 7*B*). This constitutes an immediate early response for handling the first load of cargo to be degraded before synthesis of new lysosomes can begin.

## Experimental procedures

### Reagents and antibodies

Reagents: ML-SA1 (Sigma Cat# SML0627), W7 (Sigma Cat# 681629), BAPTA-AM (Sigma Cat# A1076), apilimod (Sigma Cat# SML2974), Lysosensor Yellow/Blue DND-160 (Invitrogen Cat# L7545), Lysosensor Green DND-189 (Invitrogen Cat# L7535), FITC-Dextran (MW 10000) (Sigma Cat# FD10S), Alexa Fluor 647-Dextran (MW 10000) (Invitrogen Cat# D22914), vinblastine (Sigma Cat# V1377), Magic Red cathepsin B activity assay kit (Bio-Rad Cat# ICT937), Mirus Trans-IT LT1 transfection reagent (Mirus Cat# MIR 2300), OptiMEM (Thermo Cat# 31985070), polyethylenimine linear 25K (Polysciences Cat# 23966), doxycycline (Sigma Cat# D9891), puromycin (Sigma Cat# P8833), concanamycin A (Sigma Cat# C9705), Triton X-114 (Sigma Cat# X114), bafilomycin A1 (Sigma Cat# B1793), glycyl-L Phenylalanine 2-Naphthylamide (GPN) (Cayman Chemical Cat# 14634), Intracellular pH calibration buffer kit (Thermo Cat# P35379), protease inhibitor cocktail (Pierce Cat# A32963), Optiprep (Sigma Cat# D1556), HA magnetic agarose beads (Thermo Fisher Scientific Cat# 88837, RRID:AB_2861399), BCA Protein Assay Kit (Pierce Cat# 23227).

Antibodies: pan-cadherin (Sigma-Aldrich Cat# C1821, RRID:AB_476826) (IF: 1:300), HA (Roche Cat# ROAHAHA, RRID:AB_2687407) (WB: 1:1000), Syntaxin 7 (Proteintech Cat# 12322-1-AP, RRID:AB_2239979) (WB: 1:1000), VAMP7 (Abcam Cat# ab36195, RRID:AB_2212928) (WB: 1:1000), LAMP1 (DSHB Cat# H4A3, RRID:AB_2296838) (WB: 1:100), LAMP1 (DSHB Cat# G1/139/5, RRID:AB_10659721) (WB: 1:50), (WB: 1:1000), ATP6V1A (Abcam Cat# ab118326, RRID:AB_10899429) (WB: 1:2000), p62 (Thermo Fisher Scientific Cat# PA5-27247, RRID:AB_2544723) (WB: 1:500), alpha tubulin (Thermo Fisher Scientific Cat# 62204, RRID:AB_1965960) (WB: 1:5000), IRDye 800CW goat-anti rabbit (LI-COR Biosciences Cat# 926-32211, RRID:AB_621843), 800CW goat anti-mouse (LI-COR Biosciences Cat# 926-32210, RRID:AB_621842), 680RD goat-anti mouse (LI-COR Biosciences Cat# 926-68070, RRID:AB_10956588), IRDye 800CW Goat anti-Rat IgG (LI-COR Biosciences Cat# 926-32219, RRID:AB_1850025) (all LI-COR antibodies WB: 1:5000), Goat anti-Mouse IgG Alexa Fluor 647 (Thermo Fisher Scientific Cat# A32728, RRID:AB_2633277) (IF: 1:800).

### Cell culture and treatments

HEK-293 cells were purchased from ATCC and routinely tested for *mycoplasma* contamination. Cells were cultured in high-glucose Dulbecco’s modified Eagle’s medium supplemented with 10% fetal bovine serum at 37 °C/5% CO_2_ in a humidified incubator. Treatments: ML-SA1 was used at 25 μM in complete media for 15 min (microscopy experiments) and 30 min (biochemical experiments); 50 μM (GC-ML1 measurements). BAPTA-AM treatment was 5 μM for 3 h (biochemical experiments) and for 1.5 h (microscopy experiments) in complete media. Apilimod treatment was at 1 μM for 1 h in complete media. W7 treatment was 3 μM for 2 h in complete media.

### Plasmids and transfection

Cells were seeded in glass-bottom confocal dishes. Transfection was performed with Mirus Trans-IT LT1 reagent or PEI. Briefly, to make the DNA:lipid transfection complex, plasmid DNA and PEI (or Transit-LT1 reagent according to manufacturers’ protocol) was mixed (PEI:DNA::3:1 w/w) and incubated in OptiMEM media for 30 min at room temperature. Transfection complexes were added dropwise to cells and incubated for 24 h. The following plasmids were used in this study:

TRPML1-HA (gift from Craig Montell, Addgene plasmid # 18825; RRID:Addgene_18825), pGP-CMV-GCaMP6m (gift from Douglas Kim & GENIE Project, Addgene plasmid # 40754; RRID:Addgene_40754), pEGFP VAMP7 (gift from Thierry Galli, Addgene plasmid # 42316; RRID:Addgene_42316), pMRXIP GFP-Stx7 (gift from Noboru Mizushima, Addgene plasmid # 45921; RRID:Addgene_45921), EGFP-LC3 (gift from Karla Kirkegaard, Addgene plasmid # 11546; RRID:Addgene_11546), pmRFP-LC3, gift from Tamotsu Yoshimori (Addgene plasmid # 21075; RRID:Addgene_21075), Mucolipin1-pHcRed C1 (gift from Paul Luzio, Addgene plasmid # 62959; RRID:Addgene_62959), Mucolipin1 D471-472K-pHcRed C1 (gift from Paul Luzio, Addgene plasmid # 62961; RRID:Addgene_62961), LAMP1-mGFP (gift from Esteban Dell’Angelica, Addgene plasmid # 34831; RRID:Addgene_34831), pLJM1-Tmem192-mRFP-3xHA (gift from Roberto Zoncu, Addgene plasmid # 134631; RRID:Addgene_134631), GCaMP6m-TRPML1 (this study), pLJC5-Tmem192-3xHA (gift from David Sabatini, Addgene plasmid # 102930; RRID:Addgene_102930), tet-pLKO-puro (gift from Dmitri Wiederschain, Addgene plasmid # 21915; RRID:Addgene_21915), pMDLg/pRRE (gift from Didier Trono, Addgene plasmid # 12251; RRID:Addgene_12251), pRSV-Rev (gift from Didier Trono, Addgene plasmid # 12253; RRID:Addgene_12253), pMD2.G (gift from Didier Trono, Addgene plasmid # 12259; RRID:Addgene_12259).

### Molecular cloning

The human TRPML1 sequence was PCR amplified from TRPML1-HA using the following primers:

Forward: gagtttgtacaaatgatgacagcgaaggcggccgcaattgcccttgccaccatgaca,

Reverse: ggtatggctgattatgatctagagtcgcggccgctcaattcaccagcagcgaatgctcc.

In parallel, pGP-CMV-GCaMP6m was digested with NotI. Both the PCR product and the NotI-digested backbone were gel purified and assembled with NEBuilder HiFi DNA assembly. The following sequencing primers were used: forward: ctcttcatcgcgctcatcac, reverse: gggatgcttgatggtgtcgt. To generate doxycycline-inducible shRNA vector, Mission shRNA sequences (Sigma) were annealed and cloned between the AgeI-EcoRI sites of gel-purified tet-pLKO-puro backbone. Positive clones verified by XhoI diagnostic digest and sequencing were used for lentiviral delivery. The following shRNAs were used:

*PI4K2A*: CCTCTTCCTGAGAACACTAAC (TRCN0000195396),

*VAMP7*: GCGAGTTCTCAAGTGTCTTAG (TRCN0000298636).

### RNA extraction and qRT-PCR

Cells were grown in doxycycline-containing media for 4 to 5 days in 60 mm dishes and up to 80% confluence. Total RNA extraction was performed using Direct-zol RNA Miniprep kit according to manufactures’ protocol (Zymo Research). 0.5 to 1 μg complementary DNA were synthesized using the RevertAid First Strand complementary DNA Synthesis Kit (Thermo Scientific). Real-time PCR was performed in triplicate using SYBR Green FastMix (Quantabio) in a Rotor-Gene Q qPCR machine (Qiagen). Analysis was performed using the 2^−ΔΔCT^ method. The following primers were used:

*PI4K2A*: CGAGGCAATGACAACTGGCTGA and GCCACCTTGATAACAGGCTCCT;

*VAMP7*: GACCCTGCACTGACCCG and CGGGAACGTTCAAAATCCTCC.

### Lentiviral and shRNA work

The following plasmids were used for transfection: transfer plasmids (tet-pLKO-puro, pLJC5-Tmem192-3xHA), pMDLg, pMD2.G, and pRSV-Rev. HEK-293T cells were seeded in 10 cm tissue culture dishes to 50% confluence and transfected with the help of PEI (3:1 w/w with DNA) for 24 h. Transfection media was replaced with complete media and lentivirus was harvested after 48 h. For transduction, lentiviral media was filtered with a 0.45 μm filter, supplemented with 8 μg/ml polybrene, and added to target cells in 60 mm dishes having ∼50% confluence. Puromycin selection at 2 μg/ml was applied 48 h after transduction and stable clones were established by limiting dilution. To induce shRNA expression, cells stably expressing the shRNA constructs were kept in doxycycline media (2 μg/ml) for 72 to 96 h. Gene silencing was verified with immunoblotting/qRT-PCR.

### Quantification of lysosomal pH

Cells were seeded in glass-bottom confocal dishes. Upon reaching 50 to 60% confluence, cells were pulsed with 250 μg/ml 10 kD FITC Dextran coupled with 0.1 mg/ml 10 kD Alexa Fluor 647 Dextran in complete media overnight (16–18 h). The next day, cells were washed twice with PBS and incubated with complete Dulbecco’s modified Eagle’s medium without FITC Dextran for a 1 h chase period. Chase period was combined with any treatments. Next, the medium was replaced with a live cell imaging solution (140 mM NaCl, 2.5 mM KCl, 1.8 mM CaCl_2_, 1 mM MgCl_2_, 20 mM HEPES, pH = 7.4, sterile filtered) and cells were imaged immediately in a LSM 800 confocal system using a Plan-Apochromat 63×/1.40 Oil DIC M27 objective, excitation wavelength of 488 nm, and emission collected in the standard FITC filter. After imaging had finished, the imaging solution was sequentially replaced by a series of prewarmed pH calibration buffers (pH 7.5 > 6.5 > 5.5 > 4.5), each supplemented with 10 μM valinomycin and 10 μM nigericin. Cells were incubated for 5 min in the calibration buffer and imaged at each pH using the same field of cells and the same acquisition setting. Whole-cell ROIs were marked in Zeiss Zen Blue software for the same cell at each pH value and fluorescence intensities were recorded. Data from ≥15 cells was fitted in a standard curve using the ‘least squares’ method in GraphPad Prism v8. Experimental data was interpolated from this standard curve. For Lysosensor assays, cells were grown up to 80% confluence and then incubated with 1 μM Lysosensor Green or Lysosensor Yellow/Blue in complete media for 1 h. The cells were washed at least once with PBS and imaged under the confocal microscope.

### Measurement of TRPML Ca^2+^ release

HEK-293 cells were grown in glass-bottom confocal dishes and transiently transfected with GCaMP6m-TRPML plasmid. The next day, the medium was replaced with a low external Ca^2+^ buffer (145 mM NaCl, 5 mM KCl, 3 mM MgCl_2_, 10 mM glucose, 1 mM EGTA, and 20 mM Hepes, pH 7.4; estimated free Ca^2+^ < 10 nM) (4). Cells were incubated for 15 min in this buffer. A method described earlier for ratiometric imaging of GCaMP was followed (2). Cells were imaged with a Zeiss LSM 800 confocal unit using a Plan-Apochromat 63×/1.40 Oil DIC M27 objective. After selection of a suitable imaging area, the low external Ca^2+^ buffer was removed and imaging was started immediately in a temperature-controlled environment, followed by the addition of prewarmed 25 μM ML-SA1 dissolved in low external Ca^2+^ buffer at the 1-min mark. Images were captured sequentially by exciting with a Ca^2+^-sensitive wavelength of 488 nm and a Ca^2+^-insensitive wavelength of 405 nm, both of which were collected in the 505 to 565 nm range with a frame time of 0.63 s and a total acquisition time of 5 min. Whole-cell ROIs were marked in the Zen Blue software using the ‘Mean ROI’ function and fluorescence intensity was measured for both channels at each frame for at least five cells. The fluorescence value of background subtracted Ex:488 nm was divided by that of Ex:405 nm to obtain a ratiometric estimation of GCaMP fluorescence at each timepoint. This data was plotted against time (in seconds) using GraphPad Prism v8.

### Measurement of cathepsin B activity

Cells were grown on 12 mm coverslips up to 70% confluence. After required treatments, cells were incubated in Magic Red solution from Magic Red cathepsin B kit (Bio-Rad Cat# ICT937) at 37 °C for 30 min. This was followed by DAPI incubation (1 μg/ml) for 10 min. Cells were then mounted in Vectashield mounting media (Vector Labs Cat# H-1000-10) and images were captured by 580 nm excitation and collected in the RFP channel on a Zeiss Axioimager M2 equipped with an Apotome2 module and an ORCA Flash 4.0 LT sCMOS camera (Hamamatsu) using Plan-Apochromat 63×/1.40 oil DIC M27 objective.

### LysoIP

Following a previous protocol (56), HEK-293 cells stably expressing Tmem192-3xHA were seeded in 10 cm tissue culture dishes and grown up to 80% confluence. All subsequent steps were carried in ice with ice-cold buffers unless stated otherwise. Cells were washed twice with PBS and gently scraped in 1 ml KPBS (136 mM KCl, 10 mM KH_2_PO_4_, pH 7.25). Cell pellets were collected by centrifugation at 1000*g* for 2 min at 4 °C. This pellet was resuspended in 650 μl KPBS supplemented with 3.7% OptiPrep (Sigma) and protease inhibitors. Cells were homogenized by a two-step process: first by 35 strokes in a 2 ml Kontes glass tissue homogenizer (Kimble Chase), followed by five passes through a 25-gauge needle. The homogenate was centrifuged (1000*g*, 2 min, 4 °C) and the supernatant was collected. Six hundred microliters of this supernatant was incubated with 70 μl of prewashed HA magnetic beads (Pierce) in a microcentrifuge tube for 12 min with end-over-end mixing on a tube rotator at 4 °C. Beads were washed three times with KPBS (first wash was done with KPBS + 3.7% OptiPrep) and samples were eluted with Laemmli sample buffer by boiling.

### Immunoblotting

Cells adhered to tissue culture dishes were washed once with PBS and harvested by scraping in PBS. Cell pellet was collected by centrifugation at 400*g* at 4 °C for 5 min, which was then resuspended in an ice-cold RIPA buffer supplemented with protease inhibitors for lysis. The lysate was spun down at 14,000*g* for 10 min at 4 °C to collect the supernatant, which was then boiled with Laemmli sample buffer. Protein estimation was done with the BCA assay and equal amounts were resolved in SDS-PAGE and transferred to 0.45 μm PVDF membranes. Membranes were blocked with 5% milk in TBS or Intercept TBS blocking buffer and incubated with primary antibodies diluted in 5% milk in TBS + 0.1% Tween-20 (TBS-T) or Intercept TBS-T buffer overnight at 4 °C. The next day, they were washed with TBS-T (3 times, 5 min each) and incubated with secondary antibodies (LiCOR) in 5% milk in TBST or Intercept TBS-T blocking buffer supplemented with 0.02% SDS at room temperature for 1.5 h. After three washes with TBS-T and one wash with TBS, membranes were imaged in a LiCOR Odyssey system.

### Analysis of lysosomal SNARE complexes

Following a previously described protocol (35), cells were seeded in 10 cm tissue culture dishes and processed for LysoIP as described above. Instead of elution of total lysosomal proteins in Laemmli sample buffer, lysosomes bound to beads after the final wash were incubated with a hypotonic buffer (5 mM Tris–Cl, pH 7.0) containing protease inhibitors in ice for 10 min to elute the lysosomal luminal fraction. This luminal fraction was boiled separately in Laemmli sample buffer. After removal of the eluate, beads were incubated with 5 mM Tris–Cl supplemented with 1% Triton-X 114 and protease inhibitors to elute the total lysosomal membrane fraction. This total membrane fraction was centrifuged at 15,000*g* for 30 min at 4 °C. The supernatant was considered as the soluble membrane fraction and mixed with Laemmli sample buffer. The pellet was separately boiled in Laemmli sample buffer to generate the detergent-resistant membrane fraction. All samples were run in SDS-PAGE and analyzed with immunoblotting against syntaxin 7.

### Immunostaining

Cells were seeded in 12 mm sterile glass coverslips inside a 6-well plate. For fixation, the coverslips were washed once with PBS and incubated in 3.7% paraformaldehyde in PBS for 20 min with gentle rocking. Following this, three washes were done in PBS to remove PFA residue, and permeabilization was done with 0.2% Triton-X 100 in PBS for 10 to 12 min. Following this, blocking was done with 1% BSA + 22.52 mg/ml glycine in PBS + 0.1% Tween-20 (PBS-T) for 30 min. Primary and secondary antibody incubation was done in 1% BSA in PBS-T for overnight at 4 °C and 1.5 h at room temperature, respectively. After three washes with PBS-T and one wash with PBS (with DAPI if desired), cells were mounted in Vectashield mounting media in glass slides.

### Confocal microscopy and live imaging

HEK-293 cells were typically grown on glass coverslips or confocal dishes up to 80% confluence. Cells were imaged on a Zeiss Axio Observer.Z1 equipped with a LSM 800 confocal module using a Plan-Apochromat 63×/1.40 oil DIC M27 objective with appropriate excitation/emission filters. Typically 2× line averaging was used to improve signal-to-noise ratio, except for GFP-PI(3,5)P_2_. Same acquisition settings were used for all samples/replicates for an experiment. For live imaging, U2OS cells were seeded on confocal dishes and transfected with GFP-Stx7/GFP-VAMP7 and LAMP1-mCherry. The next day, cells were washed once in live cell imaging solution (same as the pH measurement experiments) and induced with ML-SA1 in the same solution. Images were taken in a 37 °C/5% CO_2_ environment at 1-min intervals using an Olympus IX83P1ZF widefield microscope equipped with an ORCA Fusion BT sCMOS camera (Hamamatsu) using UPLXAPO 60×/1.42 oil objective. Minor brightness/contrast adjustment to all frames was applied in Fiji for visibility.

### Image quantification

All quantifications (including Ca^2+^ measurements) were performed from data obtained from a single focal plane (typically the nuclear plane) in a confocal image, except Magic Red cathepsin assay where widefield fluorescence images were used. Same acquisition settings were used for all samples/replicates within an experiment. Binary thresholding was used to quantify the number of GFP-PI(3,5)P_2_ puncta and GFP-Stx7 puncta. Colocalization analysis was manually performed by scoring Lamp1/Syntaxin 7/VAMP7/TMEM192 puncta overlapping with LC3B. Percentage of colocalizing puncta was obtained by dividing the number of colocalizing puncta by the total number of autophagosome/lysosome puncta in that cell and converting to a percentage value. To quantify the SNARE-positive area in a lysosome, first the images were converted to micron scale using the ‘Set Scale’ function in Fiji. The ‘Freehand Selections’ tool in Fiji was used to mark the GFP-SNARE–positive area. In parallel, TMEM192-mRFP–covered circular area was measured for the same lysosome. Then the area values (in μm^2^) were divided (GFP/RFP) and converted to percentage values. Data obtained from microscopy analysis were representative for at least two independent experiments.

To measure the perinuclear index of lysosomes, we followed an earlier study (9). We quantified an average cellular area of 150 to 500 μm^2^ for HEK-293 cells. The boundary of perinuclear area was adjusted on the basis of surface area ratio with HeLa cells reported in the earlier study, from 5 μm (HeLa) to 1 μm (HEK-293, this study). LAMP intensity in this 1 μm perinuclear region and the remaining peripheral region was measured in Fiji (RRID:SCR_002285); where C1 = total LAMP signal in the cell, Np1 = 1 micron enlarged periphery around nucleus, N1 = nuclear LAMP1 signal, I_total_ = C1-N1, I_perinuclear_ = Np1-N1, I_peripheral_ = C1-Np1, I_<1_ = (I_perinuclear_/I_peripheral_)-100, I_>1_ = (I_peripheral_/I_total_)-100, and perinuclear index = I_<1_-I_>1_. More than 15 cells were measured for each sample.

### Statistical analysis

For statistical analysis of datasets, the Shapiro–Wilk test was used to assess normality of the dataset. For pairwise comparisons, unpaired Student’s *t* test was used if the dataset passed Shapiro–Wilk test (*p* < 0.05); otherwise, Mann-Whitney test was used. Typically, more than 25 cells were used for quantification, except for GCaMP measurements where three to five cells were quantified; exact N values are shown in the figures/legends. GraphPad Prism (RRID:SCR_002798) was used for all statistical analyses and for generating all plots except for the profile plots where Fiji was used. Error bars indicate SEM. ∗*p* ≤ 0.05, ∗∗*p* ≤ 0.01, ∗∗∗*p* ≤ 0.001, ∗∗∗∗*p* ≤ 0.0001, and ns *p* > 0.05.

## Data availability

All data in this manuscript is available upon request. Contact G. Juhász (juhasz.gabor@brc.hu).

## Supporting information

This article contains supporting information.

## Conflict of interest

The authors declare that they have no conflicts of interest with the contents of this article.

## Figures and Tables

**S1:**
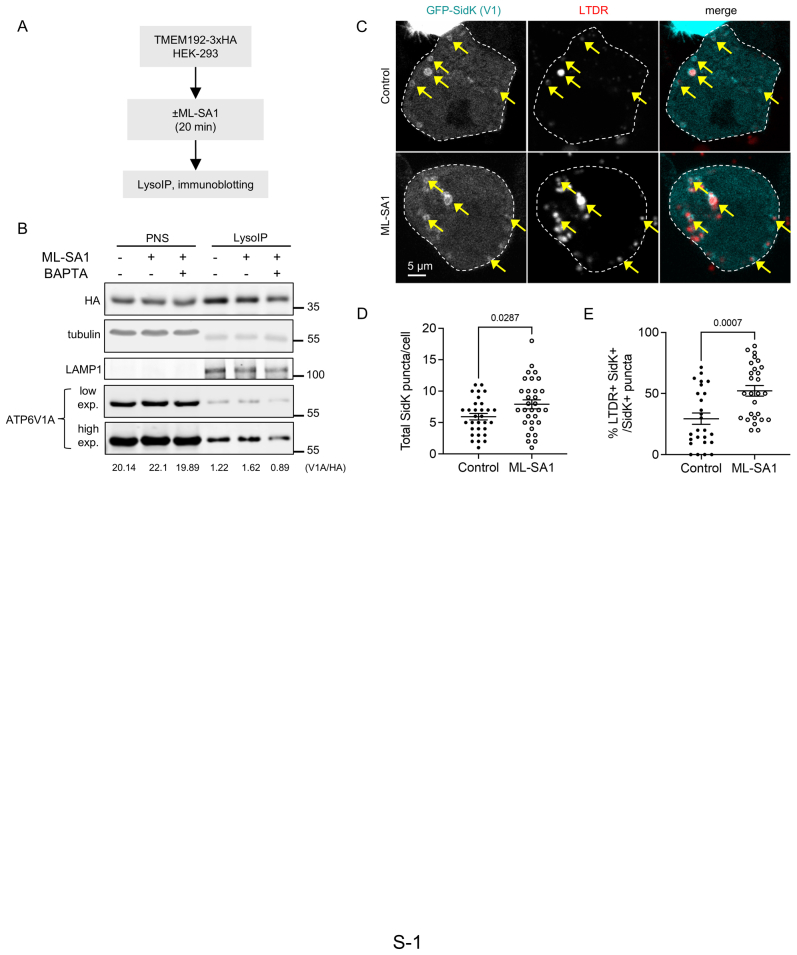


**S2:**
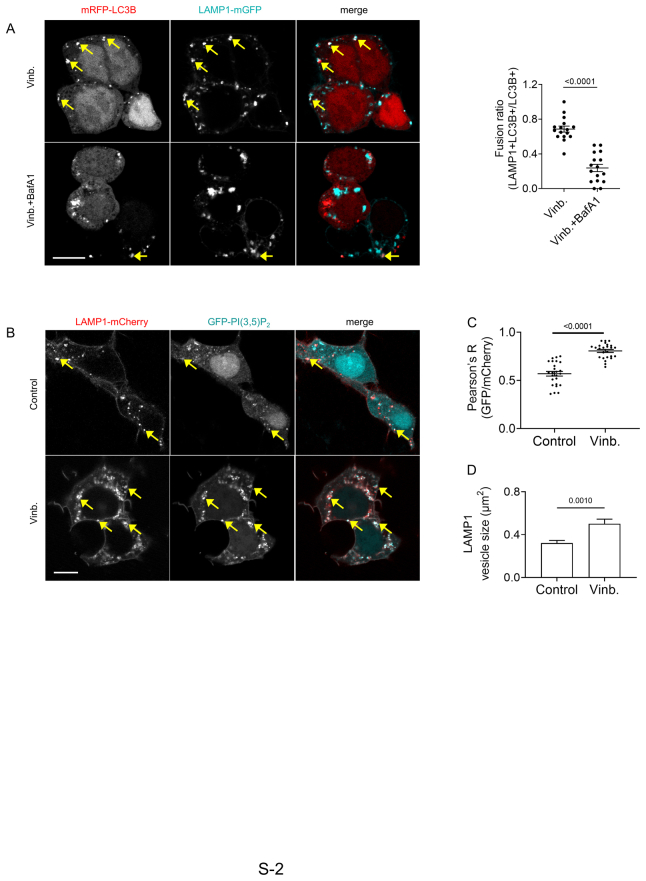


**S3:**
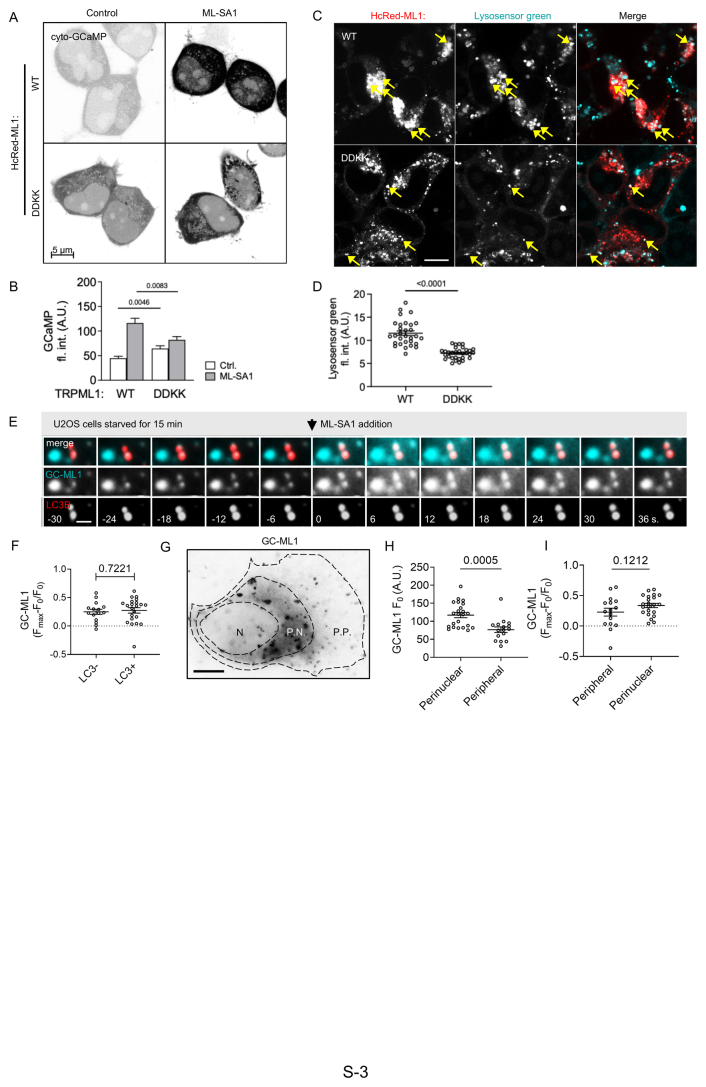


**S4:**
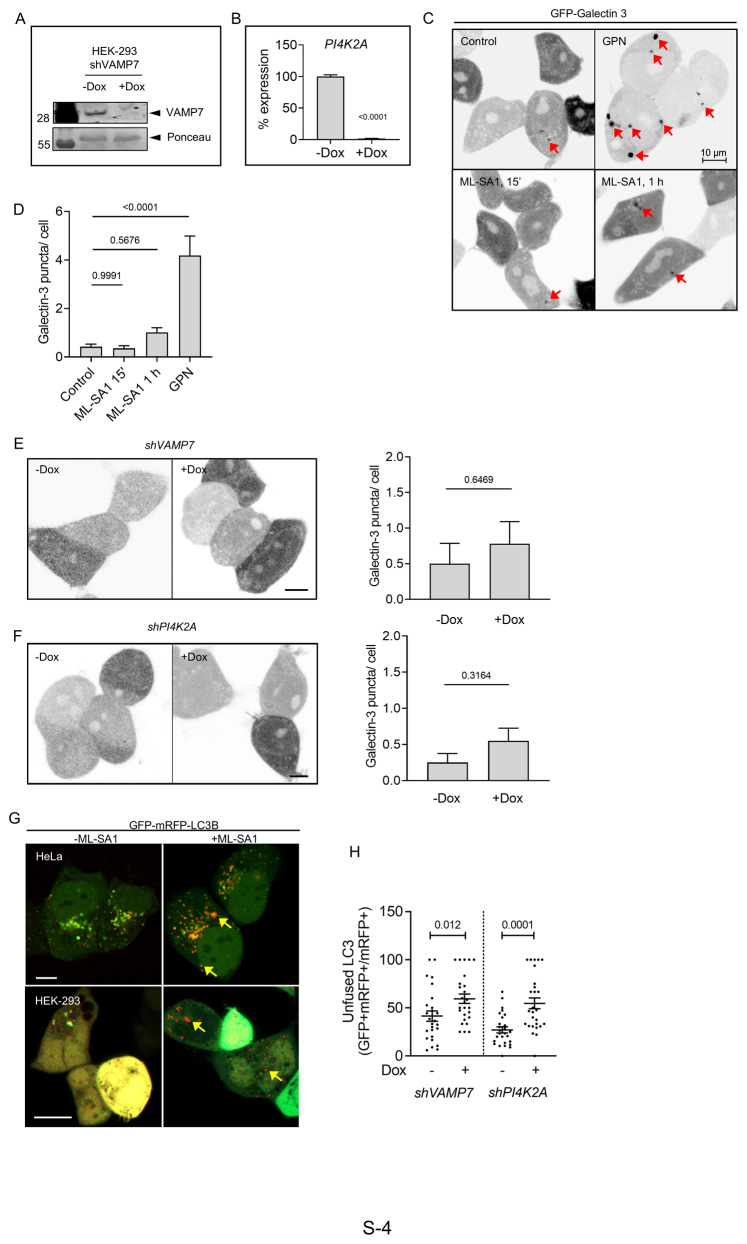


**S5:**
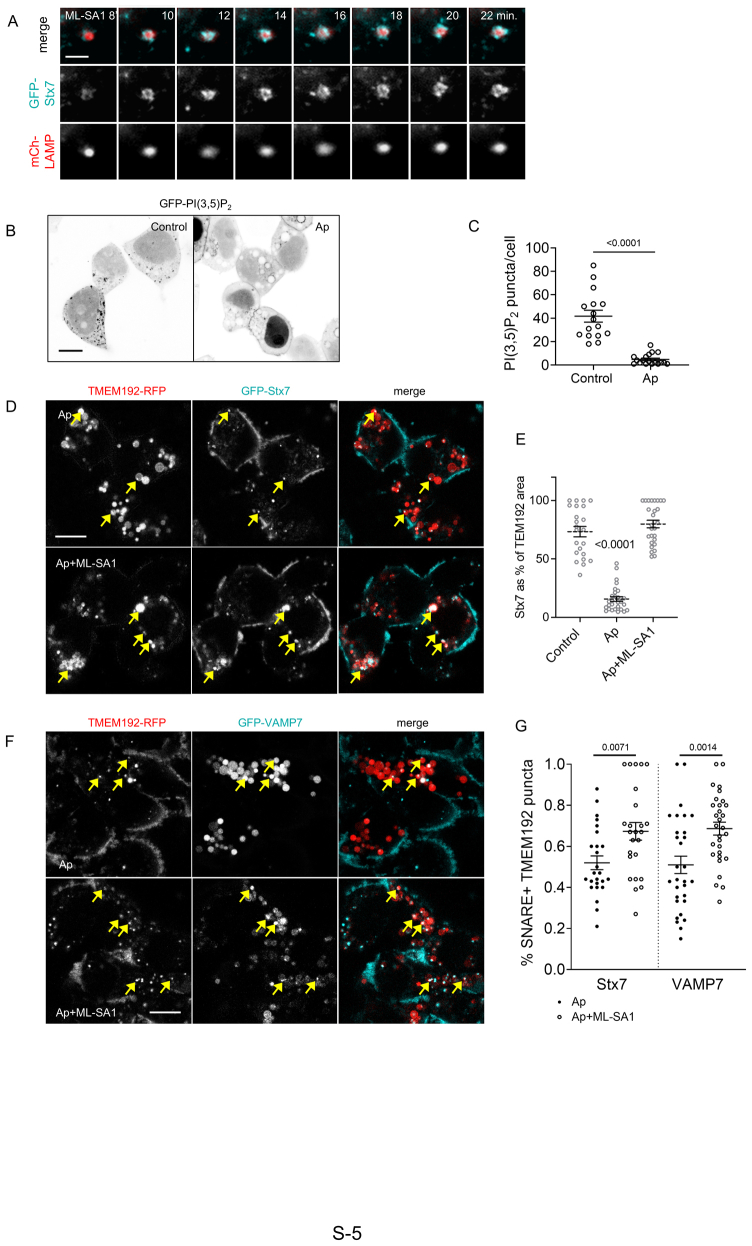


**S6:**