# Supplementary material for: Lysosomal activity depends on TRPML1-mediated Ca2+ release coupled to incoming vesicle fusions
Source: J Biol Chem. 2024 Oct 19;300(12):107911. doi: 10.1016/j.jbc.2024.107911 (PMC11599452; doi:10.1016/j.jbc.2024.107911)
Supplement: Supporting Information [file mmc1.docx]

**Supporting information**

**Lysosomal activity depends on TRPML1-mediated Ca^2+^ release coupled to incoming vesicle fusions**

Arindam Bhattacharjee^1^*^#^, Hussein Abuammar^1,2,^*, Gábor Juhász^1,3^

^1^Institute of Genetics, MTA Lendület Lysosomal Degradation Research Group, HUN-REN BRC Szeged, Szeged, Hungary

^2^Biology Doctoral School, University of Szeged, Szeged, Hungary

^3^Dept of Anatomy, Cell and Developmental Biology, ELTE, Budapest, Hungary

*shared first authors.

^#^Present address: Developmental biology group, Agharkar Research Institute, Pune, India

Correspondence to: G. Juhász ([juhasz.gabor@brc.hu](mailto:juhasz.gabor@brc.hu))

This file contains supplementary figure legends.

Supplementary figure legends

**Figure S1: TRPML1 activates mostly existing v-ATPase assemblies for acidification.**

(A) Schematic illustration of lysosomal immunopurification (lysoIP) experiments.

(B) The v-ATPase subunit ATP6V1A is only mildly enriched in TRPML-activated lysosomes during 20 minutes of ML-SA1 treatment. LAMP1: lysosome marker, tubulin: cytosol marker.

(C) Lysotracker Deep Red (LTDR) positivity of lysosomes positive for a v1-specific fluorescent reporter, GFP-SidK increases in cells treated with ML-SA1 for 20 minutes. Arrows indicate SidK positive vesicles. Single, low-expressing cells are shown.

(D) The number of v1 (SidK) positive vesicle only mildly increases during ML-SA1 treatment. N=26, Control; N=27, ML-SA1. Statistics: two-tailed Mann-Whitney u test.

(E) The LTDR positivity of SidK+ vesicles strongly increases during ML-SA1 treatment, indicating acidification (resulting in LTDR positivity) of existing v1-containing vesicles/lysosomes. N=31, statistics: two-tailed Mann-Whitney u test.

**Figure S2: Lysosomal PI(3,5)P2 enrichment and local autophagosome-lysosome fusion is promoted by the microtubule inhibitor vinblastine.**

(A) Cells were treated with vinblastine alone or together with bafilomycin A1 (100 nM). Arrows show colocalized signal indicating autophagosome-lysosome fusion during vinblastine treatment, which is disrupted in BafA1-co-treated cells (left panel). Bar: 5 μm. Right panel: fusion ratio was quantified. N=16. Statistics: two-tailed unpaired t test.

(B) Cells expressing LAMP1-mCherry and a GFP reporter for the phospholipid PI(3,5)P_2_ without or with vinblastine treatment (20 μM, 2 h). Bar: 5 μm. Arrows indicate PI(3,5)P_2_-positive LAMP1 structures.

(C) Pearson’s colocalization coefficient show that colocalization of LAMP1-mCherry and GFP-PI(3,5)P_2_ (measured from each cell) increased upon vinblastine treatment. N=25, statistics: two-tailed unpaired t test.

(D) The size of LAMP1 vesicles increased significantly upon vinblastine treatment, indicating fusion of lysosomes. N=255, control; N=200, Vinb (N refers to individual lysosomes). Statistics: two-tailed Mann-Whitney u test.

**Figure S3: Further characterization of GCaMP responses and acidification.**

(A) Cells were transfected with cyto-GCaMP to measure cytosolic calcium levels and either TRPML1 WT or DDKK, followed by ML-SA1 induction to release lysosomal Ca^2+^. Images were captured 2 minutes after treatment.

(B) Background-subtracted cellular GCaMP fluorescence was measured from (A). N=44, wt, Ctrl.; N=42, wt, ML-SA1; N=53, DDKK, Ctrl.; N=55, DDKK, ML-SA1 (N refers to individual cells). Statistics: two-tailed unpaired t tests for untreated and ML-SA1 treated sample groups, respectively.

(C) Cells were co-transfected with either HcRed-ML1 WT or HcRed-ML1 DDKK and stained with Lysosensor green to identify acidic lysosomes. Lysosensor-positive HcRed-ML1 structures (arrows) are more prominent in wt compared to DDKK, as well as clustered vs. dispersed in mutant.

(D) Whole-cell Lysosensor green fluorescence intensity was measured from cells such as in (C). N=30. Statistics: two-tailed unpaired t test.

(E) Cells expressing GCaMP-ML1 and mRFP-LC3B were starved with EBSS to induce autophagy. Afterwards, TRPML1 Ca^2+^ efflux was induced by 50 μM ML-SA1 (arrow). Bar: 2 μm.

(F) Maximal GC-ML1 response (F_max_) to ML-SA1 as a factor of basal (F_0_) TRPML1 activity was compared between individual neighboring GCaMP structures which are LC3B+ (autolyosome) or LC3B- (naïve lysosomes) from live imaging. N=15, LC3B-; N=22=LC3B+. Statistics: two-tailed unpaired t test.

(G) GC-ML1 expressing U2OS cell shows distribution of GCaMP-positive structures outlined as perinuclear (P.N.) and peripheral (P.P.). Bar: 10 μm.

(H) Peripheral and perinuclear basal GCaMP fluorescence (F_0_) was quantified. TRPML1 Ca^2+^ efflux is much higher in perinuclear lysosomes without ML-SA1 treatment. N=23, PN; N=16, PP. Statistics: two-tailed unpaired t test.

(I) The maximal GCaMP response to ML-SA1 treatment of perinuclear and peripheral lysosomes are similar. N=16, PP; N=22, PN. Statistics: two-tailed unpaired t test.

**Figure S4: Lysosomal damage response tests of experimental conditions used in this study**

(A) Cells expressing shVAMP7 were incubated with doxycycline (2 μg/ml) for 72 h. Knockdown of VAMP7 was validated by Western blotting.

(B) Cells expressing shPI4K2A were incubated with doxycycline (2 μg/ml) for 72 h. Knockdown of PI4K2A was validated by qRT-PCR, N=3 biological replicates.

(C) HEK-293 cells were transfected with GFP-Galectin 3 and treated with GPN (200 μM, 1 h) or ML-SA1 (25 μM, 15 min and 1 h). Galectin-3 positive puncta (damaged lysosomes) are indicated by red arrows. Bar: 10 μm.

(D) Quantification of lysosomal damage (Gal-3 positive puncta per cell) reveals that only GPN induces a statistically significant increase. N=105, control; N=66, GPN; N=60, ML-SA1 15 min; N=64, ML-SA1, 1 h (N refers to individual cells). Statistics: one-way ANOVA with Dunnett’s multiple comparisons.

(E, F) GFP-Gal3 transfection in cells stably expressing doxycycline-inducible shVAMP7 or shPI4K2A. Right panels show quantification of Gal-3 puncta. N=29, -Dox; N=57, +Dox (shVAMP7), N=30, -Dox; N=31, +Dox (shPI4K2A). N refers to individual cells. Statistics: two-tailed Mann-Whitney u tests. Bars: 10 μm.

(G) HeLa and HEK-293 cells were transfected with a GFP-mRFP-LC3B tandem fluorescent LC3/tfLC3 reporter. ML-SA1 treatment induces autophagic fusion (arrows), because the GFP fluorescence of this reporter is quenched in acidic organelles (e.g. lysosomes) after fusion, while mRFP still remains fluorescent: note the appearance of many red-only dots in cells after ML-SA1 treatment.

(H) Fusion ratio quantified as percentage of yellow to total red (red + yellow) puncta in GFP-mRFP-LC3B (tfLC3) expressing RNAi cells shown in Figure 4A, D. N=25 cells for all samples. Statistics: two-tailed Mann-Whitney u tests.

**Figure S5: TRPML1 activation restores abnormal SNARE distribution on apilimod lysosomes.**

(A) U2OS cells expressing GFP-Stx7 and mCherry-LAMP1 were live imaged during TRPML1 activation by 50 μM ML-SA1 as in Figure 5E. Bar: 2 μm.

(B) Cells expressing a GFP-tagged PI(3,5)P_2_ sensor were treated with apilimod for 1 h to inhibit PIKfyve activity. Punctate reporter signal is abolished from the cytoplasm after Ap treatment.

(C) Quantification of PI(3,5)P_2_ positive puncta from (B). N=16; statistics: two-tailed Mann-Whitney u test.

(D, F) Abnormal Ap-induced Stx7 (D) and VAMP7 (F) localization as small foci or in the lysosomal lumen is restored by subsequent TRPML1 activation in PIKfyve-inhibited cells. Arrows highlight the localization of GFP-SNARE positive structures on lysosomes labeled with TMEM192-mRFP. Bars: 5 μm.

(E) Stx7 fluorescence as percentage of TMEM192 area was measured from Ap and Ap+ML-SA1 treated cells as described in Materials and methods. N=23, control; N=29, Ap; N=28, Ap+ML-SA1. Statistics: Kruskal-Wallis one way ANOVA with Dunn’s post-hoc test.

(G) Quantification of data from panels D, F. The Stx7 and VAMP7 positivity of lysosomes increased after 15 minutes of ML-SA1 treatment. N=25, Stx7; N=30, VAMP7; statistics: unpaired two-tailed t tests between genotypes.

**Figure S6: Calmodulin is required for lysosome-plasma membrane recycling of Stx7.**

Cells expressing TMEM192-mRFP were transiently transfected with GFP-Stx7, and then treated with Apilimod for 1 h to inhibit PIKfyve, followed by washout in normal, ML-SA1, W7 (calmodulin inhibitor, 3 μM) or ML-SA1+W7 containing medium for 20 minutes. Afterwards, cells were fixed and immunostained for cadherin. DAPI labels nuclei (blue). Arrows indicate peripheral (cadherin-adjacent) Stx7 puncta as a proxy of Stx7 recycling post-lysosomal fusion. Bottom: quantification of peripheral (cadherin-adjacent) Stx7 puncta per cell under the conditions described. N=25, Control, Ap washout, Ap washout+W7, Ap washout+ML-SA1; N=27, Ap washout+W7+ML-SA1. Statistics: Kruskal-Wallis one-way ANOVA with Dunn’s post-hoc test.
